# Oral health status in historic population: Macroscopic and metagenomic evidence

**DOI:** 10.1371/journal.pone.0196482

**Published:** 2018-05-16

**Authors:** Claire Willmann, Xavier Mata, Kristian Hanghoej, Laure Tonasso, Lenka Tisseyre, Céline Jeziorski, Elodie Cabot, Pierre Chevet, Eric Crubézy, Ludovic Orlando, Rémi Esclassan, Catherine Thèves

**Affiliations:** 1 Laboratoire d’Anthropologie Moléculaire et d’Imagerie de Synthèse UMR 5288, Université de Toulouse, CNRS, Université Paul Sabatier, Toulouse, France; 2 Service d’odontologie de l’Hôtel-Dieu, Toulouse, France; 3 Centre for GeoGenetics, Natural History Museum of Denmark, Copenhagen, Denmark; 4 INRA, US 1426, GeT-PlaGe, Genotoul, Castanet-Tolosan, France; 5 Institut National de Recherches Archéologiques Préventives, INRAP Grand Ouest, Cesson-Sévigné, France; 6 Anthropologie Bio-Culturelle, Droit, Ethique et Santé, Faculté de Médecine Site Nord (UMR 7268), Marseille, France; University of Florence, ITALY

## Abstract

Recent developments in High-Throughput DNA sequencing (HTS) technologies and ancient DNA (aDNA) research have opened access to the characterization of the microbial communities within past populations. Most studies have, however, relied on the analysis of dental calculus as one particular material type particularly prone to the molecular preservation of ancient microbial biofilms and potential of entire teeth for microbial characterization, both of healthy communities and pathogens in ancient individuals, remains overlooked. In this study, we used shotgun sequencing to characterize the bacterial composition from historical subjects showing macroscopic evidence of oral pathologies. We first carried out a macroscopic analysis aimed at identifying carious or periodontal diseases in subjects belonging to a French rural population of the 18th century AD. We next examined radiographically six subjects showing specific, characteristic dental pathologies and applied HTS shotgun sequencing to characterize the microbial communities present in and on the dental material. The presence of *Streptococcus mutans* and also *Rothia dentocariosa*, *Actinomyces viscosus*, *Porphyromonas gingivalis*, *Tannerella forsythia*, *Pseudoramibacter alactolyticus*, *Olsenella uli* and *Parvimonas micra* was confirmed through the presence of typical signatures of post-mortem DNA damage at an average depth-of-coverage ranging from 0.5 to 7X, with a minimum of 35% (from 35 to 93%) of the positions in the genome covered at least once. Each sampled tooth showed a specific bacterial signature associated with carious or periodontal pathologies. This work demonstrates that from a healthy independent tooth, without visible macroscopic pathology, we can identify a signature of specific pathogens and deduce the oral health status of an individual.

## Introduction

Dental medicine has an active branch of research focusing on the characterization of bacteria and oral biofilms because they are associated with the most common oral pathologies: caries, periapical and periodontal diseases [1]. Such oral pathologies are extremely frequent amongst the populations of industrialized countries and have a major impact on the individual well-being and health care provisions [1, 2]. It is well established that dental plaque and calculus represent examples of microbial communities embedded within biofilms, and studies of the plaque are making a significant contribution to the understanding of these topical areas [3–5]. Dental caries consist of multifactorial diseases influenced by the host diet and are associated with increased proportions of acidogenic and aciduric (acid-tolerating) bacteria, especially from the genera *Streptococcus* (*S*. *mutans* and *S*. *sobrinus*) and *Lactobacillus*, which are involved in the enamel demineralization process [1, 4–6]. Periapical pathologies are predominantly caused by Gram positive cocci and Gram-negative rods such as *Pseudoramibacter alactolyticus*, *Olsenella uli* or *Parvimonas micra* [7–9]. In addition to causing severe local pain, periapical microorganisms can be responsible for serious complications such as cellulitis and septicemia [10]. In contrast, gingivitis is associated with a general increase in plaque mass around the gingival margin, which provokes an inflammatory response in the host, while increased levels of anaerobic bacteria, including Gram-negative proteolytic species (especially belonging to the genera *Prevotella*, *Porphyromonas*, *Tannerella*, *Fusobacterium* and *Treponema*), are recovered from periodontal pockets.

For decades, the periodontal status of ancient populations has been of interest in dental archaeology and anthropology [11]. However, the underlying studies have mostly consisted of macroscopic observations, using measurements on archeological material from various collections [12, 13]. Recently, metagenomic has provided a new tool to access the genetic information of a whole microbial community directly via the sequencing of its total DNA content [14, 15]. The application of such technologies to dental calculus material from ancient individuals has started revealing the oral microbial communities from past populations [16–19] and provides a unique opportunity to advance our knowledge on the bacterial/periodontal status of the oral cavity at key transitional periods in our history [20–22]. Yet, a number of questions still remain open, such as the exact nature of the bacteria/pathogens present at particular historical periods and whether the diversity of commensal microorganisms has been affected by modern diet and lifestyle [23]. Additionally, with only one healthy tooth available, our study attempted to reconstruct and detect ancient oral pathogens allowing the deduction of the oral health status relative to macroscopic data. To answer these questions, we investigated the oral microbiome of some 6 French individuals who lived in pre-industrial rural communities in the 18^th^ century AD.

In 2009–2010, as urban construction progressed in the city of Le Mans, in western France, archaeological graves were discovered [24, 25]. These excavations were identified as a mass burial after the battle of Le Mans, which took place between the “Catholic and Royal Army” and the “Republican Army” and was precisely dated at 12^th^-13^th^ December 1793 [24]. The graves provided a sample of 154 ancient individuals, all identified as natives from western France thanks to historical sources [26]. This archaeological group was composed mainly of young men, mostly members of the “Catholic and Royal Army”, but also included 30% of women and teenagers [27]. This sample, thus, provides a snapshot of a French rural population at the end of the 18^th^ century.

We undertook a macroscopic and molecular analysis of the Le Mans archaeological sample aimed at 1) identifying subjects with characteristic dental pathologies through complete macroscopic and radiographic examinations, 2) characterizing the taxonomic composition of the oral flora using shotgun HTS sequencing, and 3) detecting the oral pathogenic bacteria responsible for carious, periapical or periodontal diseases from an entire tooth without macroscopic pathology [28].

## Materials and methods

### Historical context, archaeological site

Following the French Revolution of 1789, the young Republic was confronted with a league of armies of monarchic Europe. In western France, military conscription was harshly resented and armed uprisings took place, later named and organized under the name of the Catholic and Royal army. This troop consisted mostly of a composite group of peasants, which represented 80% of the local population at that time. Confrontation between the revolutionary and monarchist armies resulted in the wars of Vendée [26]. On December 12^th^ and 13^th^ 1793, the two armies opposed each other in the city of Le Mans. The losses of this battle were largely to the detriment of the monarchist army, probably with some collateral victims among Le Mans inhabitants. In the days following the battle, for fear of an epidemic, the bodies were hastily and confusingly disposed of in several pits around the city.

Following the discovery of archaeological pits, the study was granted according to Orders for the prescription of a preventive archaeological operation (operation 2009–079) issued by the “Préfet de la Région Pays de la Loire”; by-law numbers: 228 and 099; Number of archaeological site or entity: 721810083 (antique site) and 72181010122 (mass graves). All specimens are publicly deposited in the “Service Régional de l’Archéologie (SRA) des Pays de Loire” which depends on the Ministry of Culture. Nine of these mass graves were excavated by the Institut National de Recherches Archéologiques Préventives (INRAP), in 2009–2010 under the Quinconce des Jacobins in Le Mans, France [24, 25]. For these nine graves, 154 skeletons were excavated, and nine subjects were analysed (213;306;307;308;309;312;403;406;702). The graves contained contextual artifacts such as a Louis XV silver crown coin, a button from an army uniform (12^th^ regiment of dragoons) and small gold crosses. Numerous skeletons showed traumatisms in correlation with the historical reports of the Battle of Le Mans [24]. For these nine graves, 154 skeletons were excavated, with 62% identified as males, 31% as females and 6% (11 remains) undetermined. Among these individuals, 87% could be considered morphologically as adults (>18 years old) and 13% as immatures. Among the immatures, 6% were adolescents (15–19 years old) and 7% infants (<15 years old) [27].

The general preservation state of the bones and dental pieces was extremely variable across individuals and graves. In particular, some of the mandibles and maxillae were almost intact, while others were fragmented (in particular at the level of very thin bone parts such as dental septa and vestibular tables), or showed more or less extensive degradation of the bony bases, sometimes going as far as complete destruction of the jaw bone.

### Sampling of teeth and bones

The teeth and bones analyzed in this work came from pits numbered from 1 to 9 (S1 Table) [27]. During the excavation, only teeth preserved within the maxillary or mandibular alveolar bone from an identified subject were sampled for DNA analysis and were directly placed in individual bags, transported to the laboratory, and stored in controlled conditions (-20°C). Photos, X-rays and Cone Beam Computed Tomography (CBCT) images of the maxillae and mandibles from selected subjects were taken in the laboratory after tooth sampling for logistical arrangements. For some individuals, bones were collected, in the same conditions as those cited above, first for individual sex identification [27] and second, for use as negative controls in shotgun sequencing.

All the laboratory work was performed in the dedicated aDNA facilities at the AMIS laboratory (Toulouse, France), according to strict aDNA standards [29, 30].

### Morphological, macroscopic and radiographic analyses

An analytical macroscopic dental study of the teeth, mandible and maxilla was performed in each of 137 subjects (89.6% of the total population), considering only teeth still positioned on dental arches [25, 31]. The dental study of all individuals was based on the macroscopic observation of the mandibular and maxillary pieces. The data obtained from the observation of the pieces were reported on an individual evaluation sheet for each subject and included tooth wear, dental calculus, carious and periodontal diseases (accessible data in [25, 32, 33]). Following this analysis, we observed serious carious, periapical or periodontal pathologies for six individuals in particular (see Table 1 and S1 Table), which were selected for a more complete investigation. Caries were diagnosed macroscopically by two observers using a dental probe and a bright light. Lesions were considered as carious if there was cavitation and a clear defect in tooth structure; enamel colorations without tooth cavitation were not taken into account. A simplified classification based on a WHO (World Health Organization) report describing different stages of carious disease was used: cavities were divided into three categories: A, B and C, depending on the severity of the lesion [34]. Category A designated enamel cavities; category B designated cavities limited to dentine and category C designated very decayed teeth, with coronary destruction and pulp communication. The number of cavities and their locations were charted (occlusal, proximal, buccal/lingual, root and pulp) [35, 36]. Additionally, X-ray examinations were performed using retroalveolar X-ray films for maxillae (X-ray apparatus: Xmind Satelec Acteon) and panoramic or occlusal X-ray films (X-ray apparatus: Kodak 3000) for mandibles.

**Table 1:** Illumina sequencing data from aDNA extracts, mapping metrics and level of contamination.

sample id	213	306	307	308	309	312	403	406	702
**Tissues**	Tooth	Tooth	*Bone*	Tooth	Tooth	*Bone*	Tooth	Tooth	*Bone*
**Total number of reads (paired-end)**	23.1 M	29.9 M	22.4 M	23.7 M	19.3 M	21.6 M	21.7 M	22.3 M	24.6 M
**Post-trimming reads**	22.9 M	29.5 M	22.2 M	23.5 M	19.2 M	21.3 M	21.5 M	22.2 M	24.4 M
**Collapsed reads**	20.9 M	20.4 M	19.9 M	22.3 M	17.9 M	14.9 M	20.5 M	19.7 M	21.7 M
Unique human reads* **(nuclear + mitochondrial)**	0.56 M	4.5 M	40 000	20 000	20 000	90 000	0.4 M	3.6 M	.
**Clonality (human)**	0.01	0.01	0.01	0.01	0.01	0.04	0.01	0.02	.
**Human nuclear genome coverage**	0.02	0.16	0.001	0.0005	0.0006	0.004	0.01	0.13	.
**Human mitochondrial genome coverage**	2.93	9.10	0.30	0.73	0.65	0.18	4.10	19.10	.
**% Endogenous**	2.5%	15.4%	0.2%	0.1%	0.1%	0.4%	1.9%	16.2%	.
**Total number of tooth pathogen reads**	122 826	280 591	1 864	961	11 576	2 490	3 593	35 143	678
**Total number of tooth pathogen reads/total number of reads (%)**	0.53%	0.94%	0.01%	0.00%	0.06%	0.01%	0.02%	0.16%	0.00%

M, millions,

* After duplicate removal, and “.” Values lower than 100 reads

Periodontal bone loss was evaluated by a visual examination using a periodontal probe and was differentiated from attrition or post-mortem damage [13, 37]. Kerr’s method using septal form and texture characteristics for assessing periodontal status was used [12, 13]. This method classifies bone septal morphology in 6 categories, which represents increasing stages of periodontal disease. According to Kerr, categories 1 and 2 represent a “healthy” periodontal state whereas categories 3, 4 and 5 represent an altered status of septal bone and suggest periodontitis (S2 Table) [12]. Dental calculus was recorded if present.

Osteolytic infectious lesions of endodontic origin, such as periapical granulomas or cysts, bone deformation due to residual periodontal cysts and intra bony cavities, were recorded by macroscopic and radiological examinations using orthopantomogram, retroalveolar X-ray or Cone Beam Computed Tomography (CBCT) techniques (X-ray apparatus: Kodak 3000) [38]. Dental abscesses were scored as present when maxillary or mandibular bone was destroyed by an infectious process creating a rounded cavity in the spongious bone and a radiolucent lesion. If the infectious phenomena were externalized, the related fistula and cortical bone loss were charted [37, 39, 40].

### Tooth and bone preparation for aDNA extraction

A well-preserved tooth was sampled for each individual. Samples were cleaned in a dedicated aDNA laboratory, applying standard precautions for working on aDNA [35, 41]. The surfaces of the teeth samples were abraded to remove the calculus when present, cleaned with bleach (at 20% for 30 sec) and rinsed with H2O MilliQ^®^ (Millipore). Each tooth or bone was exposed to UV light for 30 min on each side [27]. Tooth surfaces were abraded with single use scalpel equipment, while bones were abraded with Dremel ^®^ and samples were reduced to a fine bone powder in liquid nitrogen using a Spex SamplePrep^™^ 6870 Freezer/Mill^™^ (Fisher Scientific). DNA extraction was performed from 200 mg of tooth or bone powder, using silica filter column-based procedures, as described previously [41].

### aDNA Library preparation and sequencing

We constructed one single-indexed Illumina DNA library per individual aDNA extract (5μl), following the methodology based on blunt-ended adapter ligation (from [42, 43]), but using a NEBNext Ultra DNA Library Prep Kit for Illumina (New England Biolabs) according to the manufacturer’s protocol. Blunt-end libraries were built with 0.750 μM as the final concentration of Illumina multiplex adapters. Each library building reaction was purified on 86.5 μl of AMPure XP beads (Beckman A6388) according to the manufacturer’s protocol. Libraries were eluted by adding 25 μl TE1X following room temperature incubation for 5 min. The libraries were first amplified in a 50 μl volume reaction using 22 μl of DNA Library, 25 μl pf PCR Master Mix 2X (NEBNext Ultra DNA Library Prep Kit), 1μl of InPE1 primer (25 μM), 1 μl of InPE2 Primer (0.5 μM) and 1 μl of an Index Primer (25 μM) for which 7 nucleotides corresponded to indexing oligo sequences [42]. The first PCR cycling conditions were initial denaturation for 30 sec at 98°C, followed by 8 cycles of 10 sec denaturation at 98°C, 30 sec annealing at 60°C and 40 sec elongation at 72°C. Finally, there was a 5 min elongation step at 72°C. PCR products were purified on 50 μl of AMPure XP beads and eluted by adding 30 μl of TE1X. A second round of PCR amplification was performed from 5 μl of purified product of the first PCR in a final volume of 25 μl using 0.5U Taq Gold (Life Technologies); 1X Gold Buffer; 2 mM MgCl2; 200 μM of each dNTP; 0.1% DMSO; and 1 μl of InPE1 primer (25 μM), 1 μl of InPE2 (0.5 μM) primers [42] and 1 μl of an Index Primer (25 μM). The second PCR cycling conditions were an initial denaturation for 10 min at 92°C, followed by 8 cycles of 30 sec denaturation at 92°C, 30 sec annealing at 60°C and 40 sec elongation at 72°C, and final elongation at 72°C for 7 min. These second PCR products were purified on 30 μl of AMPure XP beads (Beckman Coulter) and eluted by adding 30 μl of TE1X. DNA contamination from the laboratory and reagents was monitored through mock controls (Extraction blank, and Library blank), which were processed at the same time as the samples.

Amplified library concentrations were estimated on a BioAnalyzer instrument using High-Sensitivity DNA chips (Agilent Technologies) for both controls and ancient samples and pooled in equimolar ratios prior to sequencing on the Illumina HiSeq2500 on the GeTPlage platform (Castanet-Tolosan, France) using 100 cycles on a paired-end mode. To evaluate possible bacterial contaminations by DNA handling (skin microbes), by laboratory sources (reagents, plastics or materials), and by storage conditions (bacteria overgrowth; [44], one Blank Extraction (BE23) and two Blank Libraries (BL19 and BL12) were amplified for 25 cycles (same conditions as for ancient samples), purified with Ampure XP beads (Beckman Coulter) to reach a concentration compatible with further sequencing. Amplified Blanks Libraries were pooled in equimolar ratios and sequenced on MiniSeq Illumina available at AMIS, using 80 cycles and a paired-end mode.

### Sequence analysis

Metagenomic sequencing was performed on six teeth from six subjects. For comparison and control of bacterial communities coming from the soil with the buccal bacterial community, three bones from three other subjects were also sequenced as environmental/soil controls (S1 Table).

The DNA sequence data generated in this study have been deposited on the NCBI database (Bioproject PRJNA302605, SRA accession numbers SRR5581849, SRR5581851-58 and SRR6785785-87). Methods for the read sequencing process, for the analysis of nuclear and mitochondrial human genomes and aDNA damage are described in S1 Appendix. The identification method of microbial communities by MetaPhlAn and MALT software are also described in S1 Appendix. Finally, dental pathogen genomes were selected for their involvement in oral pathologies such as caries, periapical abscesses and periodontal diseases according to our macroscopic and radiologic analyses. Estimates of human DNA contamination levels based on mitochondrial sequences within blanks and samples are also provided in S1 Appendix, together with an analysis of the DNA bacterial content of both Extraction and Library Blanks.

## Results

### Macroscopic and radiographic examination

**Fig 1:**
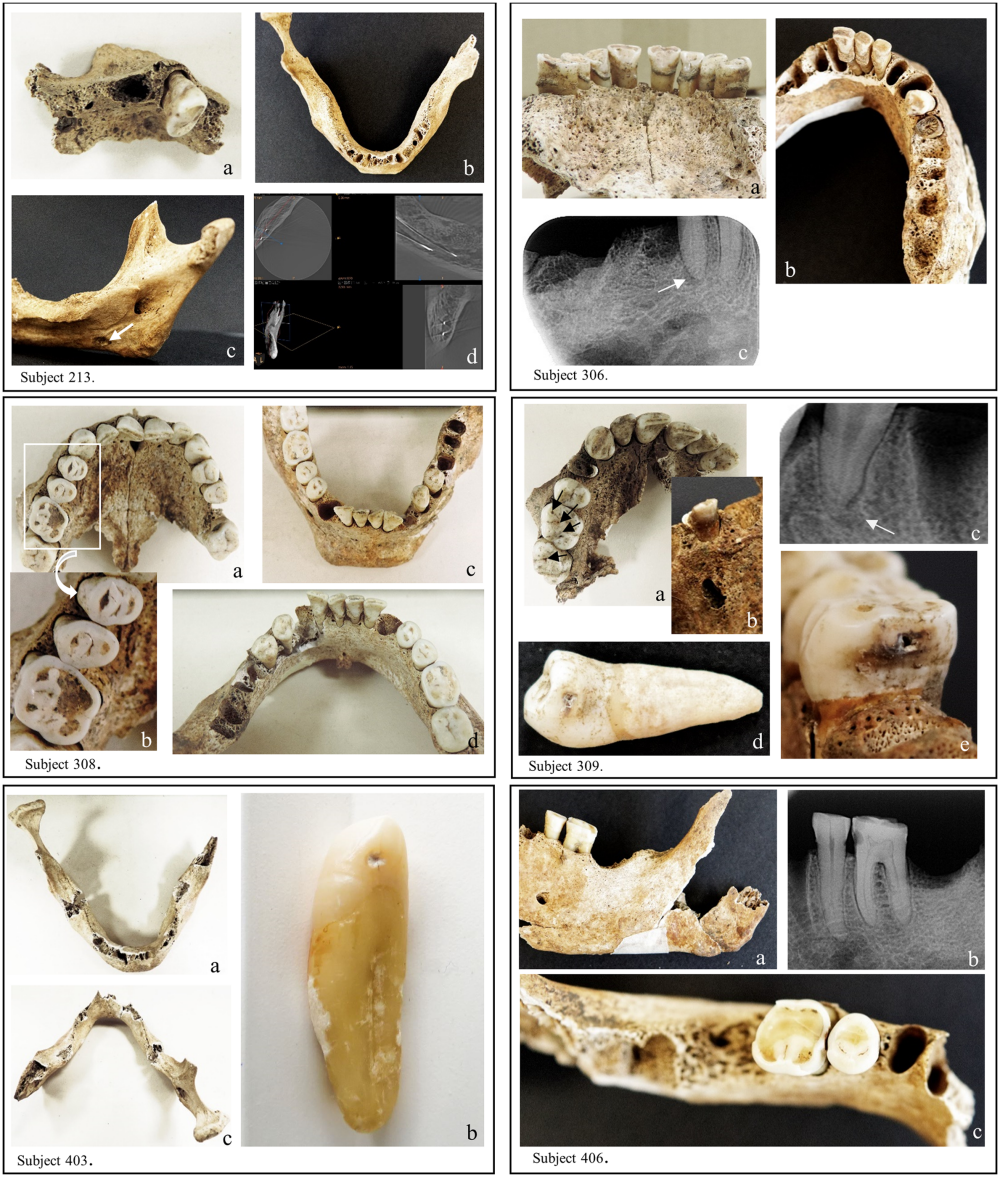
Photographs, X-ray and Cone Beam Computed Tomography (CBCT) of maxilla and mandible from sampled subjects. (Subject 213) a: maxillary septal region showing sharp, ragged aspect on both sides of tooth 14; b: deformation of the mandibular cortical bone on the right part of the horizontal branch; c: inner part of the mandibular right horizontal branch showing a fistula aperture (indicated by a white arrow); d: CBCT examination differentiating the fistula’s pathway and the inferior alveolar nerve pathway. (Subject 306) a: maxillary teeth presenting significant deposits of dental calculus; b: disorganized and riddled posterior mandibular septal morphology and destruction of the crown part of tooth 45; c: X-ray view of a granuloma on the apical part of tooth 45 (indicated by a white arrow). (Subject 308) a; c and d: sound teeth, maxillary and mandibular bones (dental calculus presents on lingual tables of teeth 31 ad 41; b: focus on important dental wear on teeth 14; 15 and 16. (Subject 309) a: groove decay on the occlusal tables of maxillary teeth 16 and 17 (indicated by black arrows); b: vestibular fenestration of the maxilla in front of the root apex of tooth 14; c: radiologically visible periapical cyst on the apex of tooth 14 (indicated by a white arrow); d: decay on the distal table of tooth 34; e: decay on the mesial table of tooth 46. (Subject 403) a and c: no teeth on the mandibular arch (except tooth 43 which was sampled for analysis); b: closer view of tooth 43 showing a small patch of distal decay. (Subject 406) a: external view of the left horizontal mandibular branch supporting teeth 35 and 36; b: retroalveolar X-ray image highlighting decay on distal table of tooth 35 and mesial table of tooth 36; c: occlusal view of teeth 35 and 36 revealing dental coloration due to the decay process between the two teeth. Pictures realized and assembled by C. Willmann.

For subject 213, the maxillary septal region in the mesial and distal sides of tooth 14 showed a sharp, ragged aspect corresponding to Kerr’s third category of periodontal disease (Fig 1, 213a).

The mandibular alveolar bone in the incisive region showed septa similar to those observed on the maxilla on both sides of tooth 14, sorted as Kerr’s third category (Fig 1, 213b). Mandibular cortical bone was deformed on the right part of the horizontal branch and a fistula aperture was present on the inner part of this branch (Fig 1, 213b and c). On CBCT examination, the fistula’s pathway was different from the inferior alveolar nerve pathway (Fig 1, 213d).

For subject 306, teeth 11 to 14 and 21 to 24 were present on the maxillary arch and had significant deposits of dental calculus on their surfaces. Septa showed a loss of normal contour with a smooth textural surface and a slightly concave form, rated as Kerr’s fourth category (Fig 1, 306a).

The septal morphology on the posterior mandibular part was disorganized, riddled and therefore charted as Kerr’s third category of periodontal disease. Macroscopically, the second lower right premolar (tooth 45) was a retained root and there was a radiologically visible granuloma on the apical part of this tooth (Fig 1, 306b and c).

For subject 308, all maxillary teeth (except tooth 26, which was on a broken part of the alveolar bone, and wisdom teeth) were present on the maxillary arch at death and were lost post-mortem. There were no traces of tooth decay, dental calculus or periodontal disease on either teeth or alveolar bone (Fig 1, 308a). Teeth 31, 32, 34, 35, 41, 42 and 44 to 47 were present on the mandibular arch. Teeth and alveolar bone appeared macroscopically sound without traces of tooth decay, or periodontal disease. Dental calculus was present on lingual tables of teeth 31 and 41 (Fig 1, 308c and d).

For subject 309, macroscopic examination of the maxilla revealed groove decay on the occlusal tables of teeth 16 and 17, charted as category A (Fig 1, 309a). Tooth 14 was a retained root and a periapical cyst formation was radiologically visible (Fig 1, 309b and c). The cyst growth had progressively destroyed both periapical spongious and buccal cortical bones, leading to a vestibular fenestration of the maxilla in front of the root apex (Fig 1, 309b). Mandibular teeth 34 and 36 were decayed on their distal tables and therefore classified in category B (Fig 1, 309d). Decays were also observed on mesial tables of teeth 36 and 46, charted as category B (Fig 1, 309e).

For subject 403, there were no teeth on the mandibular arch except tooth 43, which presented a small area of decay (category A) on its distal surface (Fig 1, 403a, b and c).

For subject 406, macroscopic and radiological examinations of the mandible highlighted tooth decay on the mesial table of tooth 36 and on the distal table of tooth 35, rated as category B, without signs of dental pulp necrosis or periapical lesions (Fig 1, 406a, b and c). The results section of S1 Appendix provides a more detailed morphological analysis of the six selected subjects.

### Endogenous aDNA

The metagenomic data obtained from aDNA extractions of whole teeth and bone fragments from ten subjects are described in Table 1. We observed that, while numbers of reads obtained from each library were between 19.3 and 29.9 million (M), the number of reads mapping against the human nuclear and mitochondrial genome was very variable between subjects and tissues. This pertains to marked differential preservation levels in the material analyzed, despite its relatively limited age.

**Fig 2:**
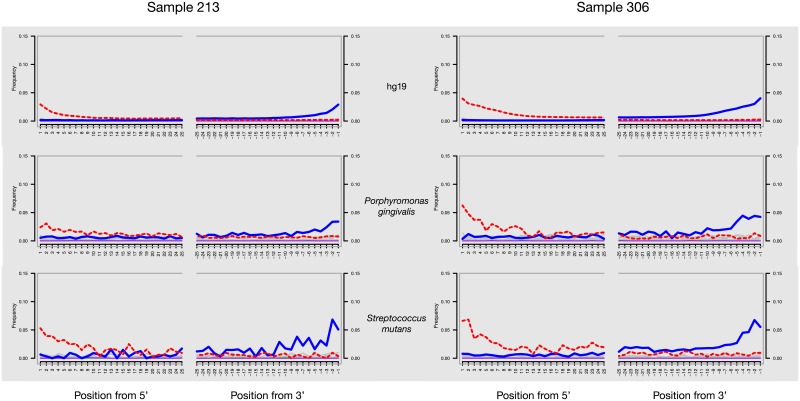
DNA damage patterns for teeth of subjects 213 and 306. The frequencies of all possible mismatches observed between the human nuclear genome (hg19), the *P*. *gingivalis* and *S*. *mutans* chromosomes and their mapped reads, respectively, are reported in gray according to the distance from 5’ end (left panel, first 25 nucleotides sequenced) and distance to 3’end (right panel, last 25 nucleotides sequenced). The typical DNA damage mutations C>T (5’) and G>A (3’) are reported in the dotted and solid lines, respectively.

The authenticity of ancient human DNA was confirmed through the presence of typical molecular signatures of post-mortem DNA damage (Fig 2 and S2 Appendix), including fragmentation patterns consistent with depurination and mis-incorporation patterns supporting cytosine deamination within overhangs [45]. This analysis was applied on all samples, when the number of reads was sufficient, for nuclear DNA (hg 19) and main oral pathogens (see S2 Appendix). It appears that seeing the observed transitions for certain species, the mapping could be less specific and could cluster reads from common genus. MtDNA and bacterial analyses on samples and Blanks showed the absence of contamination, see results on Endogenous DNA in S1 Appendix for more details.

### Metagenomic profiling

**Fig 3:**
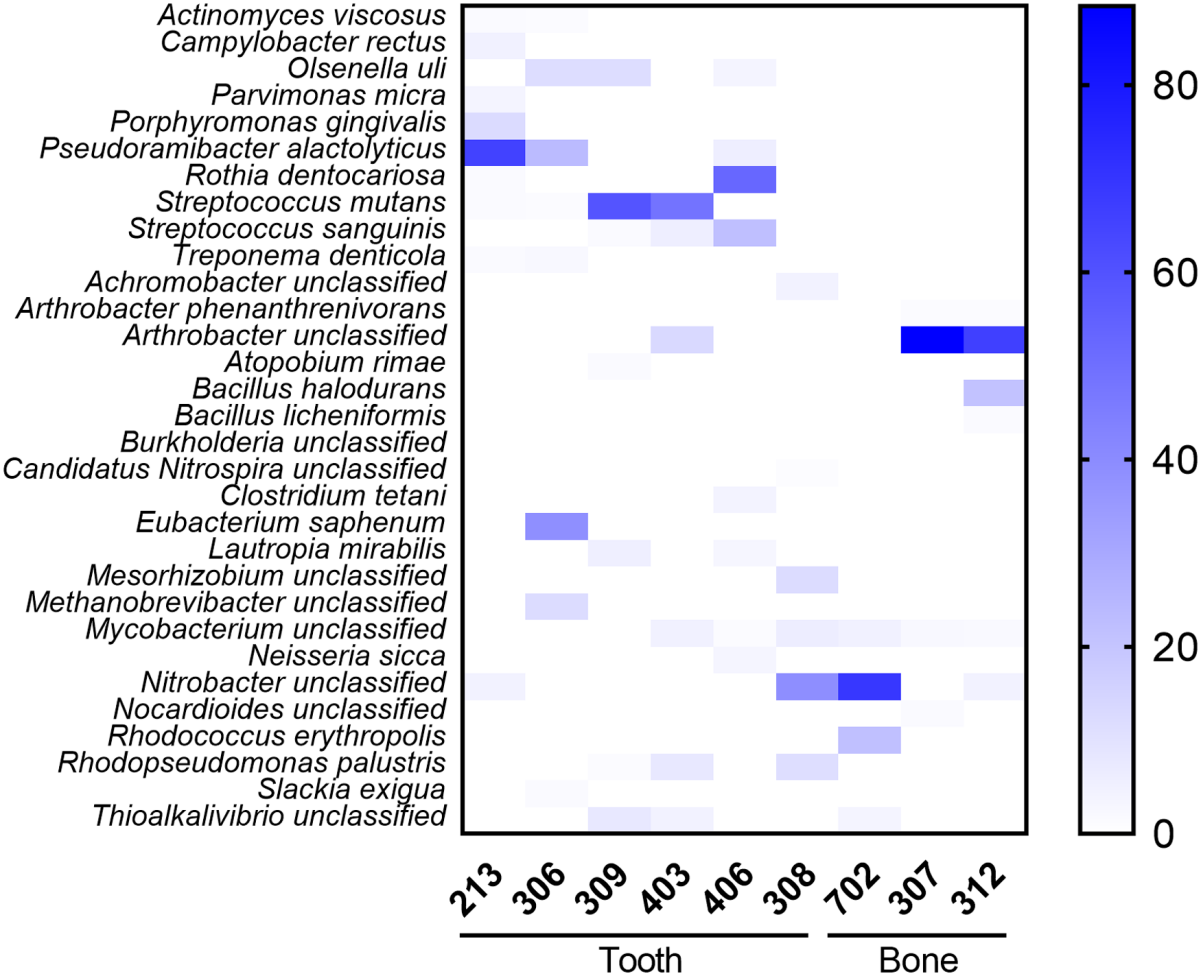
Microbial analyses of ancient samples using MetaPhlAn. Heat Map representation of microbial taxonomic composition for teeth and bones libraries was realized with GraphPad Prism v.7 (GraphPad; La Jolla, CA).

Microbial taxonomic profiling was performed using both the methodology described by Schubert and colleagues [46] and based on the MetaPhlAn specific database, and MALT [47] applied to the NCBI nucleotide database (https://www.ncbi.nlm.nih.gov/nucleotide). One output of MetaPhlAn software was the relative abundances of reads showing significant matches to microbial genome databases. Fig 3 illustrates specific microbial abundances (%) detected in MetaPhlAn database in teeth and bones libraries. It was noted that the bone libraries 312 and 307 were different from 702, with a specific pattern demonstrating no contamination by bacterial pathogen DNA in the laboratory steps. Bone libraries were essentially composed of bacteria from the soil: mainly of *Arthrobacter sp*. for sample 307; of *Nitrobacter sp*. and of *Bacillus haludorans* for sample 312; of *Nitrobacter sp*. and of *Rhodococcus erythropolis* for sample 702. The MetaPhlAn analysis of the bacterial composition of tooth libraries showed the presence of dental pathogens (Fig 3): *Actinomyces viscosus*, *Campylobacter rectus*, *Olsenella uli*, *Parvimonas micra*, *Porphyromonas gingivalis*, *Pseudoramicter alactolyticus*, *Streptococcus mutans*/*sanguinis*, *Treponema denticola*, and *Rothia dentocariosa*, which were identified in subjects 213, 306, 309, 403, and 406. No dental pathogens were detected using both MetaPhlAn and MALT in the tooth of subject 308, suggesting both a healthy tooth and the absence of cross-contamination between samples.

Differences were observed between the taxonomic read assignments derived from MetaPhlAn and MALT, especially pertaining to the identification of three oral bacteria (*P*. *alactolyticus*, *C*. *rectus* and *A*. *viscosus*), which were identified in MetaPhlAn (from 48% to 0.4% in S4 Table for 213, 306 and 406 samples in S5 Table for percentage edited by MetaPhlAn) but not in MALT (S6 Table). Reciprocally, MALT revealed the presence of the dental pathogen *Tannerella forsythia* in 213 sample at a particularly-high abundance level (34%) and in sample 306 (9%; S4 and S6 Tables with complete hits from MALT-MEGAN6), but this pathogen remained undetected by MetaPhlAn. This confirms that different computational tools presently available for carrying out a taxonomic identification of past microbial communities show different performances, probably due to various sensitivity and specificity levels, as well as differences in their underlying database [44].

**Fig 4:**
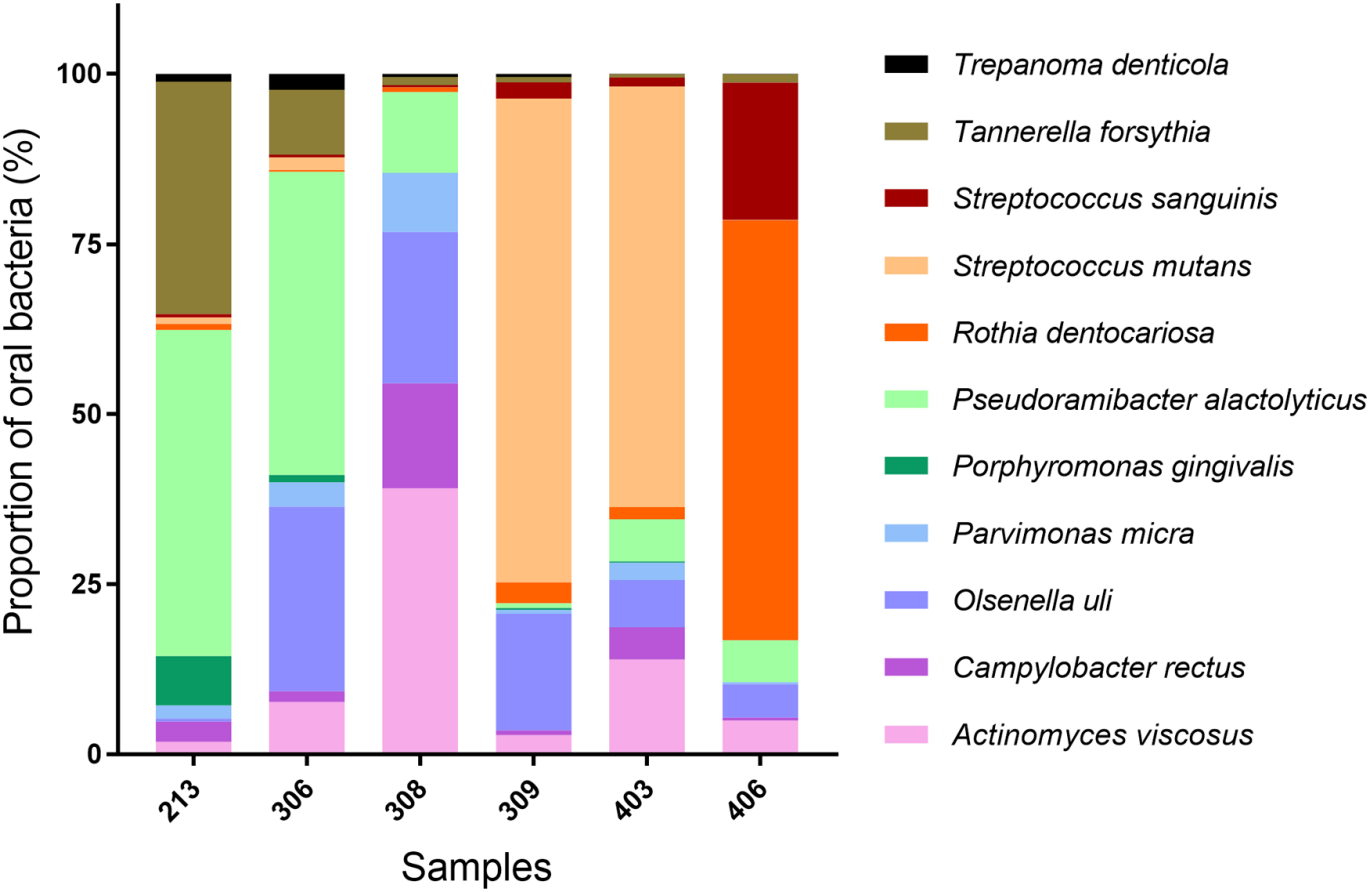
Graphical representation of 11 dental pathogens per tooth sample highlighting specific bacterial composition. Graphical representation was realized with GraphPad Prism v.7 (GraphPad; La Jolla, CA).

In order to confirm the taxonomic assignments to major dental pathogens, we mapped the sequences against 11 reference genomes of dental pathogens involved in oral diseases like carious, periapical and periodontal processes [8, 48–56], listed in S3 Table. As shown in Table 1, the number of sequences showing high-quality alignments against pathogenic bacterial genomes differed amongst the tissues and subjects analyzed. Subjects 309 and 403 showed reads mapped against practically only one bacterial species, *S*. *mutans* (S4 Table); subject 406 presented a high number of reads for *R*. *dentocariosa* and reads mapped against *S*. *sanguinis*; subjects 213 and 306 gave some specific, substantial sequences characteristic of a pathological oral microbiome, such as *P*. *gingivalis* and *P*. *alactolyticus* in high numbers. Concerning these dental pathogens, we noticed that 1) high coverage was found for several pathogen genomes, from 0.5 to 7X. For example, in samples 213 and 306, we found *P*. *alactolyticus* at 4.4X and 7X, *T*. *Forsythia* at 2X and 0.98X respectively; in 306, *O*. *uli* at 4.8X; in 406, *R*. *dentocariosa* at 1X; in 213, *P*. *gingivalis* at 0.6X and, in 309, *S*. *mutans* at 0.5X; 2). The mapped sequences covered positions in the genome at least once, with a minimum of 35%: from 35 to 50% for *P*.*gingivalis*, *R*. *dentocariosa* and *S*. *mutans*, 75% for *T*. *forsythia*, 82% for *P*. *alactolyticus*, and 93% for *O*.*uli*; 3) a specific pathogen signature was associated with the tooth of a given individual as seen in the detailed S4 Table and Fig 4, which gives a more accurate indication of the number of pathogen sequences (in bold) associated with the DNA extracted from the tooth of each subject. All these results were confirmed by displaying them with the IGV software, which showed a uniform distribution of reads along the bacterial chromosomes.

## Discussion

Ancient oral microbiomes have only been recently opened to biomolecular investigation [57]. Here, we combined macroscopic and radiologic analyses with metagenomic analyses to identify dental pathogens present in bacterial communities from past individuals (Figs 1, 3 and 4). In contrast to previous studies, our methodology relied on taxonomic profiles recovered from total aDNA available from an entire healthy tooth (including root, pulp and cementum) and was thus not limited to dental calculus. HTS read mapping helped to identify characteristic pathogens responsible for carious, periapical or periodontal diseases [58] in six individuals that lived in the late 18^th^ century (S1 Table), including not only *S*. *mutans*, but also *R*. *dentocariosa*, *A*. *viscosus*, *P*. *gingivalis*, *P*. *alactolyticus*, *O*. *uli*, *T*. *forsythia* and *P*. *micra* (S4 Table).

Interestingly, we detected a strong association between carious teeth from subjects 309 and 403 and the presence of *S*. *mutans* in the genetic data. This bacterium was identified some decades ago as the main etiological agent associated with the initiation of dental caries [1, 54]. Traditional culture-based methods have shown that *S*. *mutans* can be considered as the ‘chief pathogen’ for dental caries initiation [59]. It is also generally accepted that there is a relationship between *S*. *mutans* and diet, with individuals having frequent carbohydrate consumption showing increased levels of cariogenic bacteria such as *S*. *mutans* and a greater risk for dental caries development [4, 5, 60]. *S*. *mutans* is also considered as the organism best-adapted to a cariogenic environment (high sugar/low pH) [4, 5].

Metagenomic analysis of the tooth sampled from subject 406 also showed high levels of *S*. *sanguinis* (S4 Table and see Fig 4). *S*. *sanguinis* is a gram-positive, facultative anaerobe bacterium involved in carious disease [61]. *S*. *mutans* and *S*. *sanguinis* counteract each other in the process of oral biofilm formation, as *S*. *sanguinis* is able to inhibit *S*. *mutans* development [55]. Interestingly, subject 406 also revealed the presence of *R*. *dentocariosa*, representing as much as 62% of the bacterial species identified. To the best of our knowledge, this is the first time that *R*. *dentocariosa* is identified in an ancient population in such amounts (S4 Table; Fig 4). This bacterium is a commensal aerobic and facultative anaerobic, gram-positive organism showing both coccoid and branched filament elements [51, 53, 62]. It was previously known as *A*. *dentocariosa* [63], and was first isolated from carious dentin in humans. It is mostly found in the flora of the oral cavity, dental caries, and dental plaque from periodontal patients, but also in blood, respiratory secretions, abscesses, and wounds [53, 62, 64]. *R*. *dentocariosa* has long been considered as a low-virulence bacterium in humans but its potential to cause clinically-significant infections in immunocompromised patients is increasingly acknowledged [65]. This opportunistic pathogen is mainly involved in inflammatory processes and can induce opportunistic infections with an oral starting point, such as infective endocarditis, septicemia and pneumonia amongst others [52– 54, 65–67].

It is interesting to observe that, in both subjects 213 and 306, *P*. *gingivalis* and *T*. *forsythia* were identified together with *T*. *denticola*, another bacterial pathogen. These obligate anaerobic Gram-negative bacteria form the periodontal “red complex” [51, 68, 69], a complex secreting virulence factors, where the three bacteria act synergistically, ultimately leading to the inflammation of the host periodontal tissue, bone immuno-inflammatory resorption and chronic periodontitis and other forms of periodontal disease [70]. (S4 Table; Fig 4) [68, 71]. Interestingly, both subjects 213 and 306 showed strong morphological evidence of ante-mortem dental losses. For subject 306, there was a large dental calculus deposit on the maxillary teeth (Fig 1, 306a) and, for subject 213, tooth 14 revealed periodontal bone loss rated as Kerr’s third category, which suggests an area undergoing an acute burst of periodontal activity and bone resorption consistent with the activity of the “red complex” (Fig 1, 213a). In contrast, septa from the maxillary bone of subject 306 showed a loss of normal contour with a smooth textural surface and a slightly concave form, which, according to Kerr, could correspond to quiescent non-progressive periodontitis (Fig 1, 306a).

We also found molecular signatures of a group of bacteria involved in endodontic troubles in four (213, 306, 309 and 406) of the six individuals analyzed. In particular, samples 213, 306 and 406 revealed the presence of *P*. *alactolyticus*, an anaerobic Gram-positive rod [72] considered by some authors as a good candidate for participation in the etiology of different forms of periradicular diseases [52, 58]. Moreover, it is amongst the most frequently identified micro-organism in the root canal of necrotic teeth associated with acute periapical abscesses [73, 74]. Macroscopically, the mandibular bone of subject 213 was deformed on the right part of the horizontal branch, which is reminiscent of an endosseous infectious phenomenon (Fig 1, 213b). On radiological and CBCT examinations, it suggested a residual periapical cyst in the right molar part of the mandibular bone, whose stoma was draining through an accessory canal of the mandibular nerve (Fig 1, 213d). For subject 306, tooth 45 was a retained root and there was a radiologically visible granuloma on its apical part (Fig 1, 306b and c). This tooth may have progressively decayed and broken down, leading to pulp necrosis and periapical granuloma formation. There were no periapical infections identified on teeth from subject 406 but the maxilla was missing, the mandibular bone was damaged and numerous teeth had been lost post-mortem, all of which impeded the diagnosis of possible endodontic lesions.

Subjects 306 and 309 were positive for *O*. *uli*, a Gram-positive anaerobic rod which has been recently recognized as a member of the endodontic microbial consortium of teeth with apical periodontitis. It is found in the common microbiota associated with primary endodontic infection [50, 75]. Both on macroscopic and X-ray examinations, subject 309 showed a large abscess on the apical part of tooth 14, which had progressively destroyed the maxillary bone, leading to a vestibular fenestration in front of the root apex (Fig 1, 309b and c). As was the case for tooth 45 on subject 306, the tooth seemed to have progressively decayed and broken down causing pulp inflammation and necrosis.

Finally, the Gram-positive anaerobic coccus *P*. *micra* has been detected in subjects 213 and 306 [76]. This bacteria is known as part of the normal flora of the oral cavity and is extensively recognized as an oral pathogen. It has been isolated from multiple polymicrobial infections such as periapical and endoperiodontal lesions, periapical abscesses and periodontitis [8, 76, 77]. It has also long been recognized as a putative endodontic pathogen in necrotic root canals by studies using bacterial culture and molecular methods [78]. Macroscopic and radiological analysis of these two subjects showed endodontic and periapical impairments, in line with the molecular data (Fig 1, 213b, c, d and 306b and c).

The possibility of describing oral health, microbiomes and the evolution of oral pathogens from teeth, bones and dental calculus has been already established [16–18, 79, 80]. Our study confirmed that opportunistic pathogens associated with carious, endodontic and periodontal diseases can be identified in ancient tooth material. Moreover, in this work, we proposed a method to open access to the oral health status of an individual based on one healthy tooth, without calculus or macroscopic signs of disease. A specific microbial signature is associated to each subject which can help to diagnose oral pathologies in ancient dental human remains in absence of physiological evidence of ailments, for example, when parts of the jaw bones are missing.

From a historical standpoint, the 18th century AD in France was considered as a major period in the evolution of dental hygiene and increased lifespan of the dentition [81, 82]. This was also a period of progress in global hygiene [83]. According to a meta-analysis study collecting data from 29 cohorts with 4998 individuals [84], the frequency of caries and ante-mortem tooth loss was relatively stable prior to the 18th century, and mainly evenly distributed across Europe. But, the increasing availability of sugar would have led to a rise in caries and tooth loss from the 18th century onwards. Future work applying the methodology presented here to the full temporal range may provide direct evidence for this hypothesis. Applying the methodology described here to dental remains may also help understand the intimate role of the oral microbiome in the development of oral diseases.

## Supporting information

S1 Table Description of the 9 ancient human samples from archaeological graves resulting of the Battle of Le Mans, on 12th-13th December 1793. (XLSX)

S2 Table Classification suggested by Kerr, representing the evolution of periodontal disease. (XLSX)

S3 Table List of bacterial genomes used for mapping. (XLSX)

S4 Table Alignment statistics of reads mapped against dental pathogen genomes in teeth DNA extracts. (XLSX)

S5 Table Estimation of the percentage of reads mapped against known reference genomes by MetaPhlAn. Subsample created for estimation of the percentage of reads mapped against known reference genomes by MetaPhlAn to compare with Blank Extraction and Libraries.
(XLSX)

S6 Table A RMA result file produced by MALT for analysis in MEGAN6. (XLSX)

S1 Appendix Supplementary data on M&M and results. (DOC)

S2 Appendix Map Damage results for all samples. (PDF)
