# Supplementary material for: Oral health status in historic population: Macroscopic and metagenomic evidence
Source: PLoS One. 2018 May 16;13(5):e0196482. doi: 10.1371/journal.pone.0196482 (PMC5955521; doi:10.1371/journal.pone.0196482)
Supplement: S1 Appendix — (DOC) [file pone.0196482.s007.doc]

**Oral health status in historic population: Macroscopic and metagenomic evidence**

Claire Willmann^1,2¶^, Xavier Mata^1¶^, Kristian Hanghoej^1,6^, Laure Tonasso^1^, Lenka Tisseyre^1^, Céline Jeziorski^3^, Elodie Cabot^4,5^, Pierre Chevet^4^, Eric Crubézy^1^, Ludovic Orlando^1,6^, Rémi Esclassan^1,2&^,Catherine Thèves^1*&^

**S1 Appendix**

^1^ Laboratoire d’Anthropologie Moléculaire et d’Imagerie de Synthèse UMR 5288, Université de Toulouse, CNRS, Université Paul Sabatier, Toulouse, France.

^2^ Service d’odontologie de l’Hôtel-Dieu, 4 rue Viguerie 31062 Toulouse, France.

^3^ INRA, US 1426, GeT-PlaGe, Genotoul, Castanet-Tolosan, France.

^4^Institut National de Recherches Archéologiques Préventives, INRAP Grand Ouest, 37 rue du Bignon, 35577 Cesson-Sévigné, France.

^5^ Anthropologie Bio-Culturelle, Droit, Ethique et Santé. Faculté de Médecine Site Nord (UMR 7268), 51 Boulevard Pierre Dramard, 13 344 Marseille Cedex 15, France.

^6^Centre for GeoGenetics, Natural History Museum of Denmark, Øster Voldgade 5-7, 1350K Copenhagen, Denmark

^#^ Corresponding author

Email: [catherine.theves@univ-tlse3.fr](mailto:catherine.theves@univ-tlse3.fr)

^¶^ These authors contributed equally to this work.

^&^ These authors also contributed equally to this work.

**Supplementary material and methods**

**Sequence analyses**

Briefly, Illumina sequencing reads were trimmed for adapter sequences using AdapterRemoval [1]. Paired-end reads overlapping for at least 11 nucleotides were collapsed and were then treated as single-end reads. Trimmed reads and collapsed reads were aligned against the Homo sapiens reference genome hg19 and the Homo sapiens complete mitochondrial genome sequence (revised Cambridge Reference Sequence; GenBank Accession no. NC_012920.1) [2] using BWA version 0.7.10 r789 [3] with the parameters suggested by Schubert *et al.* [4]. Only reads with a mapping quality higher than 25 were selected. PCR duplicates were removed using the MarkDuplicates function of PICARD TOOLS version 1.88 (http://picard.sourceforge.net) and the SAMtools view utility [5], as described by Schubert *et al.*[4].

An analysis of ancient DNA (aDNA) damage was performed using the mapDamage package, version 2.0.1 [6, 7], which first computes misincorporation patterns from NGS data sets and then incorporates a statistical model of postmortem aDNA damage to estimate degradation parameters using a Bayesian framework. Default settings were used with the option –forward (50 end only) on the BAM alignment files [5] resulting from the mapping of each human genome reference (hg19) data set and main oral pathogens when the number of reads was sufficient (*Streptococcus mutans,* *Porphyromonas gingivalis, Actinomices viscosus, Pseudoramibacter alactolyticus, Rothia dentocariosa, Streptococcus sanguinis and Tannerella forsythia*).

Despite the implementation of strict DNA protocols, it is difficult to completely avoid contamination from modern DNA when working with ancient human material - in particular when dealing with samples that have been handled previously during excavation. Accurate estimates of contamination levels require large amounts of genomic data, so we restricted this analysis to samples with a mitochondrial genome coverage above five times (5X) here. We used the mtDNA-Server [8] to perform a contamination check based on the current phylogeny in order to avoid misinterpretations and erroneous conclusions. This tool provides an analysis of human mitochondrial DNA data, currently focusing on reliable identification of heteroplasmy (>= 1%) and contamination. The analysis of DNA contamination was investigated for 2 samples (subjects 306 and 406) where we had sufficient data (9X and 19X of coverage respectively). In case of contamination caused by different mtDNA sequences, the profiles lead to different valid haplogroups, and heteroplasmic variants are divided into major and minor profiles, which are segregated into different branches in cases of contamination.

To complete the analysis of the potential mitochondrial contamination, we sequenced one Blank Extraction (BE23) and two Blank Libraries (BL12 and BL19). Between 1.2 and 2 millions of paired-end reads were produced for each Blank Extraction and Blank Libraries and we investigated the presence of human mitochondrial sequences following the same protocol as samples (see above).

Microbial communities present in the extracts were profiled using MetaPhlAn (Metagenomic Phylogenetic Analysis version 1.7.7, February 2013) and MALT tool (MEGAN Alignment Tool version 0.4.0) [9, 10]. For MetaPhlAn, we mapped the collapsed reads (Table 1) from each sample to the markers of the MetaPhlAn database using the default parameters of the BOWTIE 2 version 2.1.0 aligner [11] and a sensitive global alignment strategy (default end-to-end mode) for read mapping. To avoid a bias in the calculation of the microbial relative abundance, PCR duplicates were identified using a python script stripped down from Paleomix [12]. Then, using the MetaPhlAn program, microbial taxonomic groups and their relative abundance were determined at all taxonomic levels (S5 Table). To conclude, the metagenomic visualization, we used GraphPad Prism 7 Software.

For MALT screening, first we used the malt-build to construct a MALT index on the NCBI nucleotide database, January 2018 (<https://www.ncbi.nlm.nih.gov/nucleotide>) using the default parameters except for the step size option at 4 (-step) to allow the indexing of this huge database with 1To of ram memory. Then, we aligned the collapsed reads (Table 1) of all samples on this database using malt-run in semi-global mode. The RMA file produced by MALT was used for interactive analysis of taxonomic composition in MEGAN6 (S6 Table).

In order to evaluate possible bacterial contamination by lab procedures [13] we performed a MetaPhlAn analysis with the same method on Blanks. This analysis revealed only three and unidentified bacterial sequences in BE23 Blank and no sequences in two Blank Libraries (BL12 and BL19). To verify if these results were not linked to the low number of produced reads in comparison with the samples, 1.2 millions of reads were randomly selected using the “seqtk sample” command (seqtk sample -s100 read1.fq 1200000 > sub1.fq and seqtk sample -s100 read2.fq 1200000 > sub1.fq) for all samples to create subsamples and the same MetaPhlAn analyses was performed (see S5 Table, subsample sheets).

Then, reads were aligned with the reference sequences of 11 dental pathogen genomes (S3 Table) using BWA version 0.7.10-r789 [3] with the parameters suggested by Schubert *et al.* [4]. Only reads with a mapping quality higher than 25 were kept. PCR duplicates were removed using the MarkDuplicates function of PICARD TOOLS version 1.88 and the SAMtools view utility [5]. The Integrative Genomics Viewer (IGV) [14, 15], a high-performance visualization tool, was used to explore the mapped data on the genome of pathogenic dental bacteria. To perform a proportion of bacterial genomes found in tooth samples GraphPad Prism 7 Software was used.

**Results**

**Macroscopic and radiographic examination**

For subject 213, there was only one fragment of the maxilla supporting the upper right first premolar, tooth 14. The mandibular bone was well preserved except on the left condyle area. Teeth 34, 35, 37, 38, and 45 to 48 had suffered ante-mortem loss while teeth 31 to 33, 36 and 41 to 44 were lost post-mortem. Healing and new bone formation were observed on the posterior parts of the mandible.

For subject 306, only the anterior part of the maxilla was available. The mandibular cortical bone showed a fracture on its left ramus and body parts, caused by a cold steel stroke. The alveolar bone was intact. Teeth 33 to 35, 42 and 43 were lost post-mortem whereas there was ante-mortem loss of mandibular posterior teeth 36 to 38 and 46 to 48, with traces of alveolar bone healing. Teeth 31, 32, 41, 44 and 45 were present on the mandibular arch and retro-incisal dental calculus was found in large quantities on the lingual tables of teeth 31, 32 and 41.

For subject 308, an important attrition phenomenon was observed, especially on teeth located on the right side of the maxillary arch (Fig 1, 308b). The mandible showed post-mortem fractures and post-mortem dental losses of teeth 33, 36, 37 and 43.

For subject 309, teeth 11 to 17 and 21 to 23 were on the maxillary arch. The posterior left part of the maxillary bone was missing. There were no traces of periodontal bone loss on either the maxilla or the mandible. Mandibular teeth 33, 37, 38 and 43 had suffered post-mortem loss. The other mandibular teeth (31, 32, 34 to 36, 41, 42 and 44 to 48) were present on the arch.

For subject 403, the maxilla was missing. Cortical and alveolar bone were actually damaged in the posterior areas of the mandible. Teeth 31, 32, 34, 35, 41, 42, 44 and 45 had been lost post-mortem whereas teeth 33, 36 to 38 and 46 to 48 were lost ante-mortem; bone healing was visible at the posterior part of the mandible. Periodontal bone loss was not observed.

For subject 406, the maxilla was absent. The mandibular bone was damaged, mostly on its anterior part, teeth 31, 32 and 41 were missing as was the case for teeth 47 and 48 on its posterior right part. Teeth 37 and 38 had suffered ante-mortem loss; bone remodeling and healing processes were underway. There were no objective signs of periodontal disease on this mandible. Teeth 33, 34 and 42 to 45 were lost post-mortem whereas teeth 35, 36 and 46 were still present on the mandibular arch and showed deep marks of dental wear.

**Endogenous aDNA**

The analysis of mtDNA contamination was investigated for 2 samples (subjects 306 and 406) and based on the identification of heteroplasmy (>= 1%) and therefore contamination, the mtDNA-Server tool did not show the presence of minors haplogroups for these 2 samples suggesting no mtDNA contamination, confirming that our results were not driven by modern human contaminant mtDNA.

Concerning the mitochondrial analysis of BL23, BE12 and BE19, only three sequences were found in BE23. These sequences were blasted against all mitochondrial sequences of our samples and no sequence with 100% of identities was found.

In order to evaluate possible bacterial contamination by lab procedures, a MetaPhlAn analysis was performed on BE23, BL12, BL19 and on the 1.2 million reads of subsamples. This analysis revealed only three unidentified bacterial sequences in BE23 Blank and no sequences in two Blank Libraries (BL12 and BL19) and also confirmed the identification of main oral bacteria, with sufficient number of reads, for subsamples (see S5 Table, subsamples sheet).

**References**

1. Lindgreen, S., AdapterRemoval: easy cleaning of next-generation sequencing reads. BMC Res Notes, 2012. **5**: p. 337.

2. Andrews, R.M., et al., Reanalysis and revision of the Cambridge reference sequence for human mitochondrial DNA. Nat Genet, 1999. **23**(2): p. 147.

3. Li, H. and R. Durbin, Fast and accurate short read alignment with Burrows-Wheeler transform. Bioinformatics, 2009. **25**(14): p. 1754-60.

4. Schubert, M., et al., Improving ancient DNA read mapping against modern reference genomes. BMC Genomics, 2012. **13**: p. 178.

5. Li, H., et al., The Sequence Alignment/Map format and SAMtools. Bioinformatics, 2009. **25**(16): p. 2078-9.

6. Ginolhac, A., et al., mapDamage: testing for damage patterns in ancient DNA sequences. Bioinformatics, 2011. **27**(15): p. 2153-5.

7. Jonsson, H., et al., mapDamage2.0: fast approximate Bayesian estimates of ancient DNA damage parameters. Bioinformatics, 2013. **29**(13): p. 1682-4.

8. Weissensteiner, H., et al., mtDNA-Server: next-generation sequencing data analysis of human mitochondrial DNA in the cloud. Nucleic Acids Res, 2016. **44**(W1): p. W64-9.

9. Segata, N., et al., Metagenomic microbial community profiling using unique clade-specific marker genes. Nat Methods, 2012. **9**(8): p. 811-4.

10. Herbig, A., et al., MALT : fast alignment and analysis of metagenomic DNA sequence data applied to the Tyrolean Iceman. BioRxiv : 050559., 2016.

11. Langmead, B. and S.L. Salzberg, Fast gapped-read alignment with Bowtie 2. Nat Methods, 2012. **9**(4): p. 357-9.

12. Schubert, M., et al., Characterization of ancient and modern genomes by SNP detection and phylogenomic and metagenomic analysis using PALEOMIX. Nat Protoc, 2014. **9**(5): p. 1056-82.

13. Warinner, C., et al., A Robust Framework for Microbial Archaeology. Annu Rev Genomics Hum Genet, 2017. **18**: p. 321-356.

14. Robinson, J.T., et al., Integrative genomics viewer, in Nat Biotechnol. 2011: United States. p. 24-6.

15. Thorvaldsdottir, H., J.T. Robinson, and J.P. Mesirov, Integrative Genomics Viewer (IGV): high-performance genomics data visualization and exploration. Brief Bioinform, 2013. **14**(2): p. 178-92.
