# Supplementary material for: Oral health status in historic population: Macroscopic and metagenomic evidence
Source: PLoS One. 2018 May 16;13(5):e0196482. doi: 10.1371/journal.pone.0196482 (PMC5955521; doi:10.1371/journal.pone.0196482)

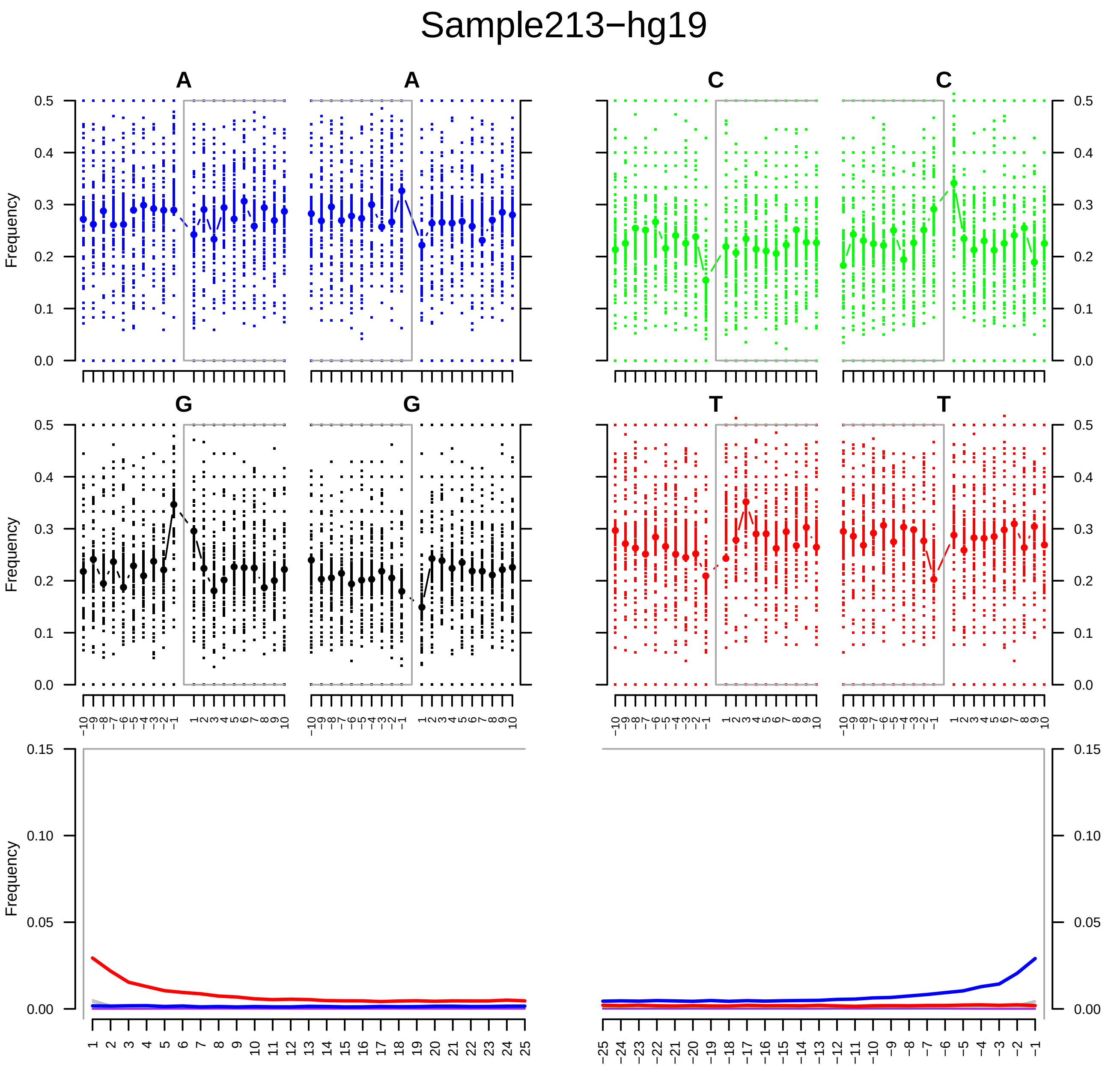

Sample213−hg19
A
A
C
C
G
G
T
T
Frequency
0.0
0.1
0.2
0.3
0.4
0.5
0.00
0.05
0.10
0.15

# Sample306−hg19

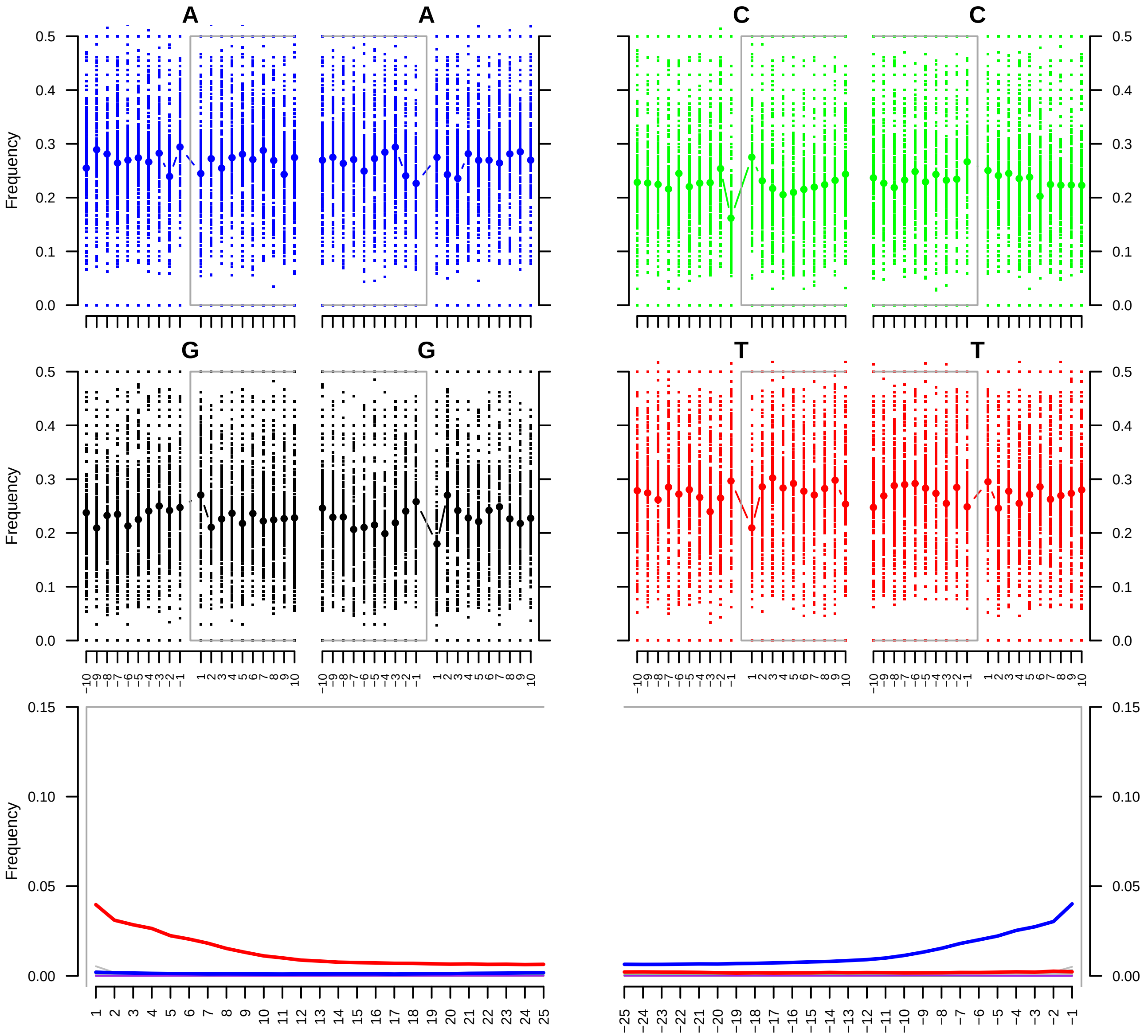

# Sample307−hg19

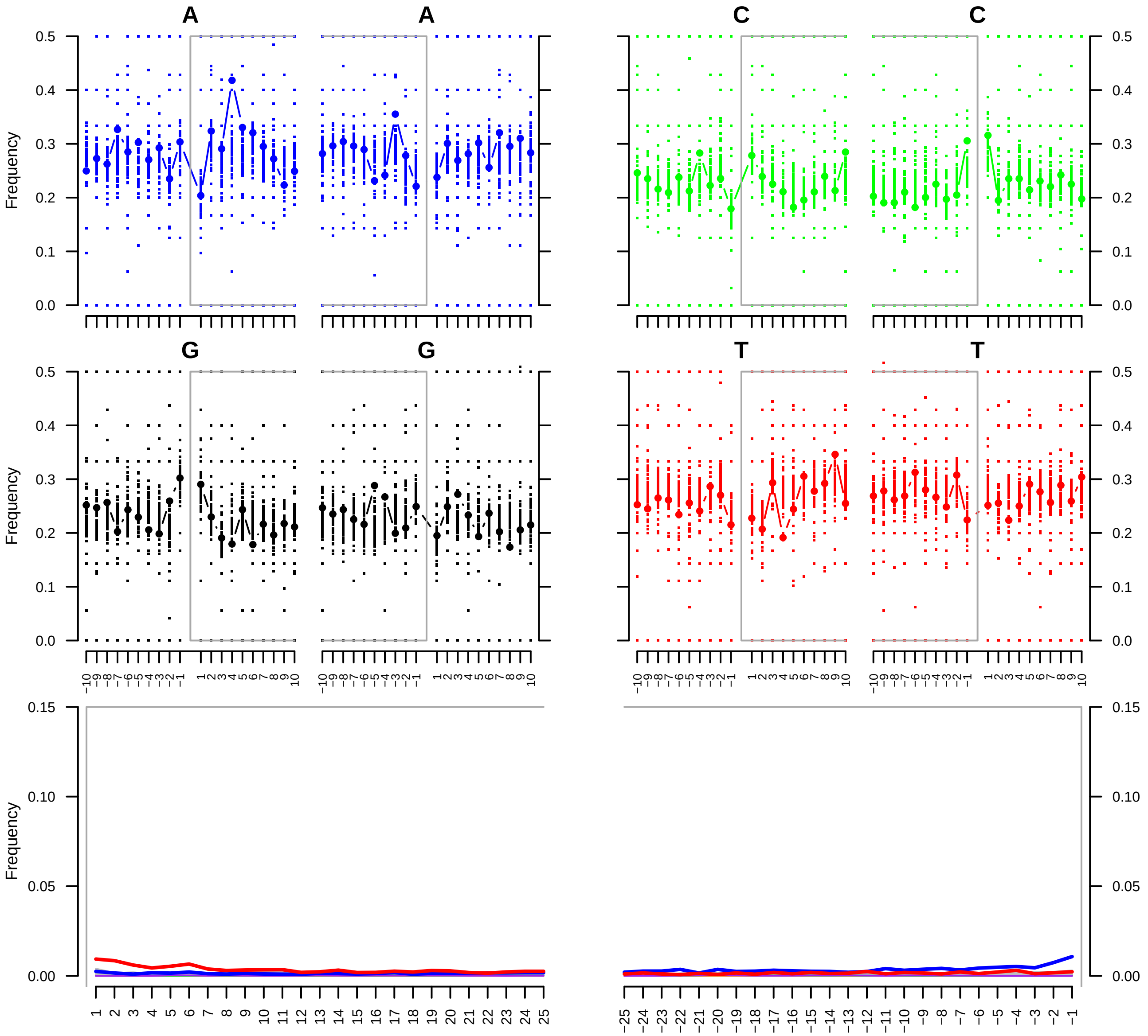

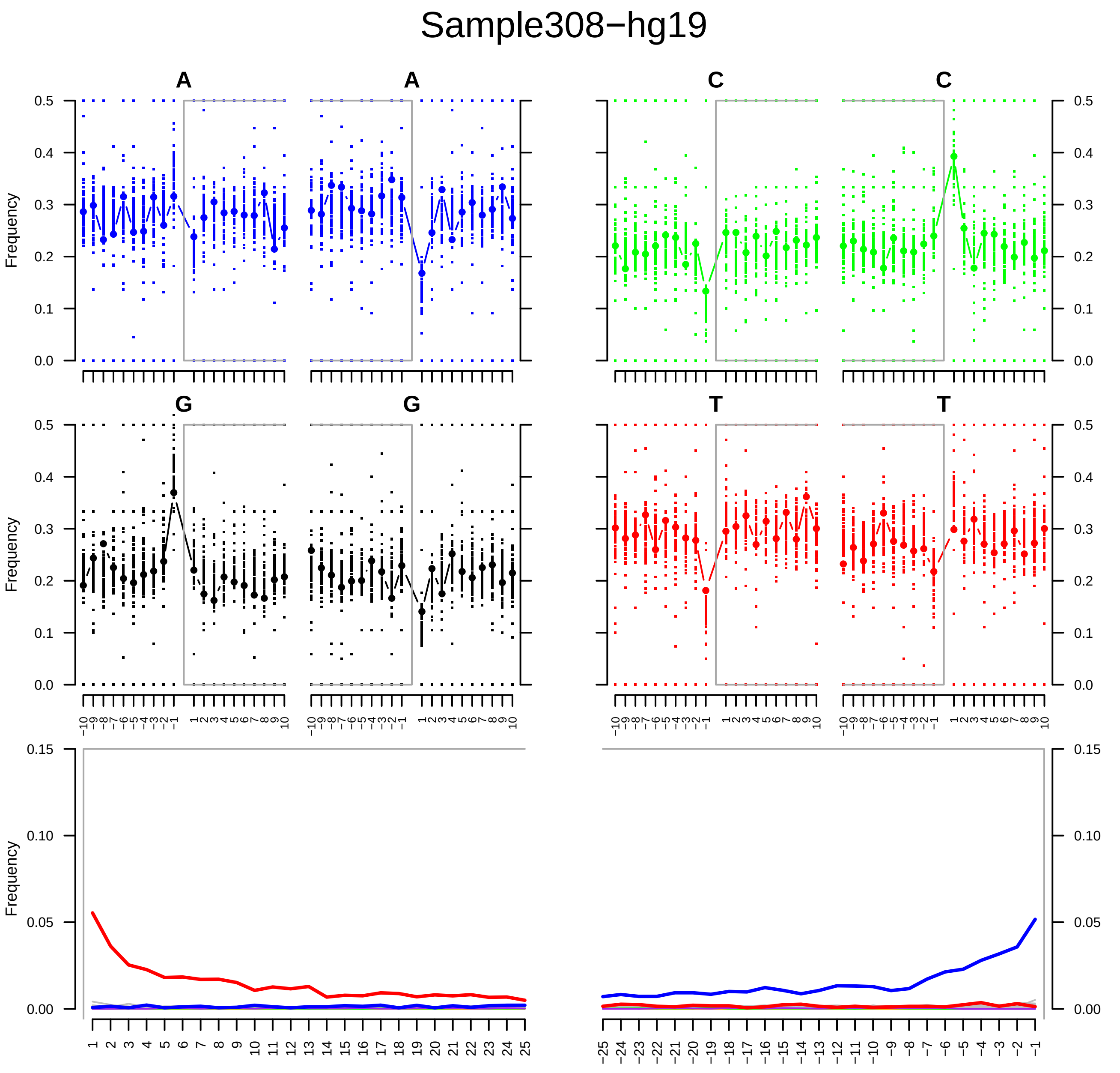
Sample308−hg19
A
A
C
C
G
G
T
T
Frequency
Frequency
Frequency
0.0
0.1
0.2
0.3
0.4
0.5
0.00
0.05
0.10
0.15

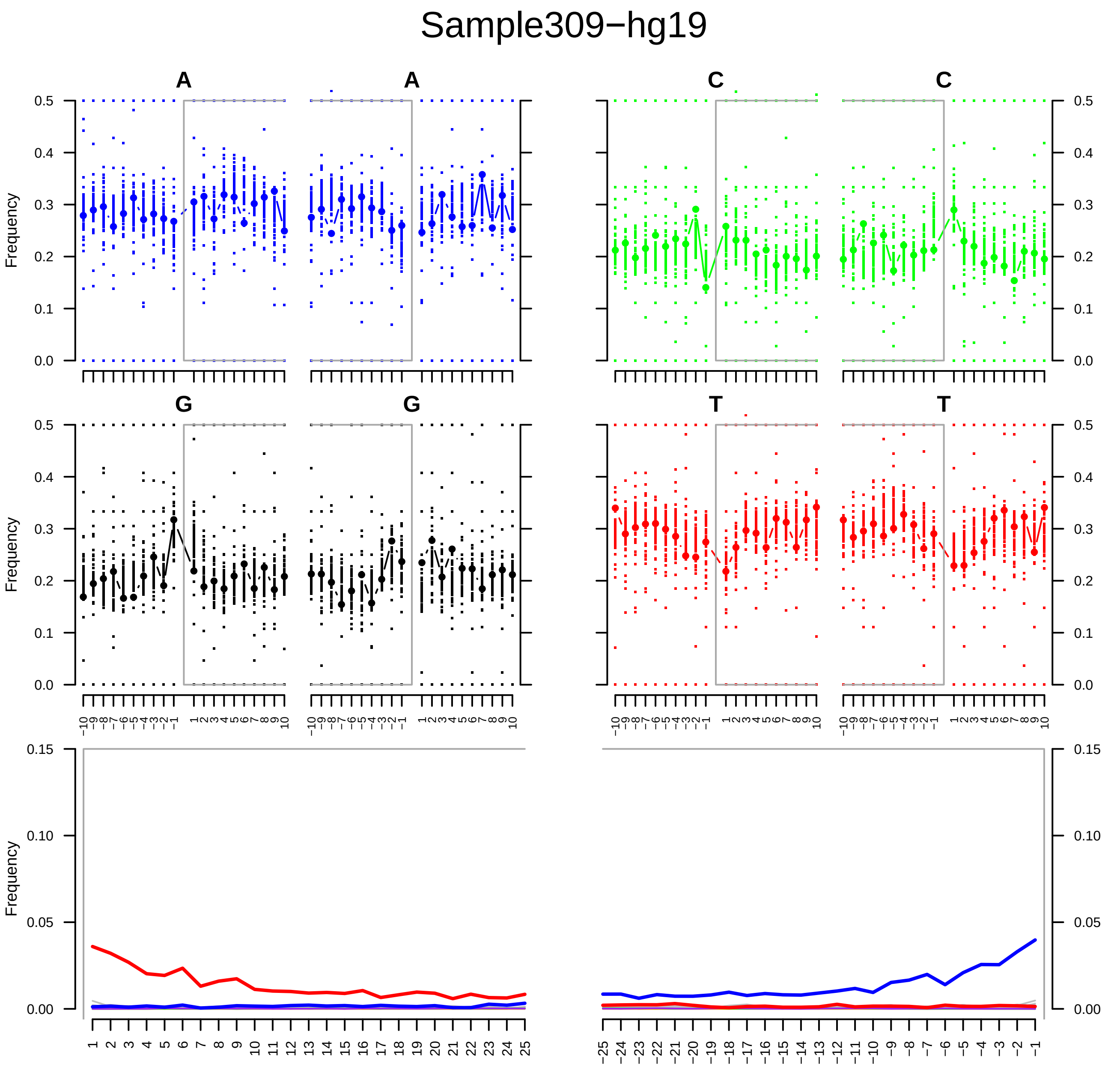

Sample309−hg19
A
A
C
C
G
G
T
T
Frequency
Frequency
Frequency
0.0
0.1
0.2
0.3
0.4
0.5
0.00
0.05
0.10
0.15
−10 −9 −8 −7 −6 −5 −4 −3 −2 −1 1 2 3 4 5 6 7 8 9 10
1 2 3 4 5 6 7 8 9 10 11 12 13 14 15 16 17 18 19 20 21 22 23 24 25
−25 −24 −23 −22 −21 −20 −19 −18 −17 −16 −15 −14 −13 −12 −11 −10 −9 −8 −7 −6 −5 −4 −3 −2 −1

# Sample312−hg19

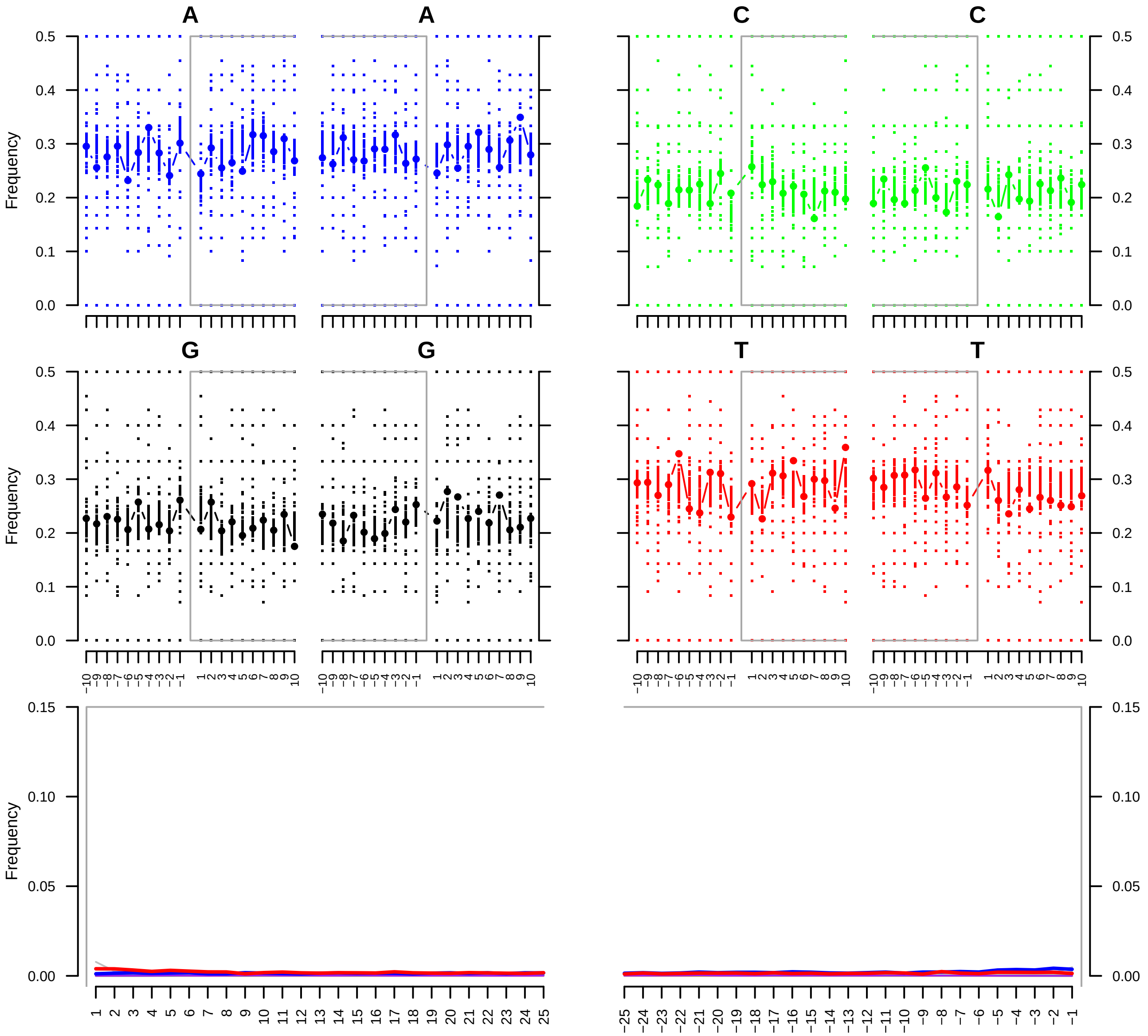

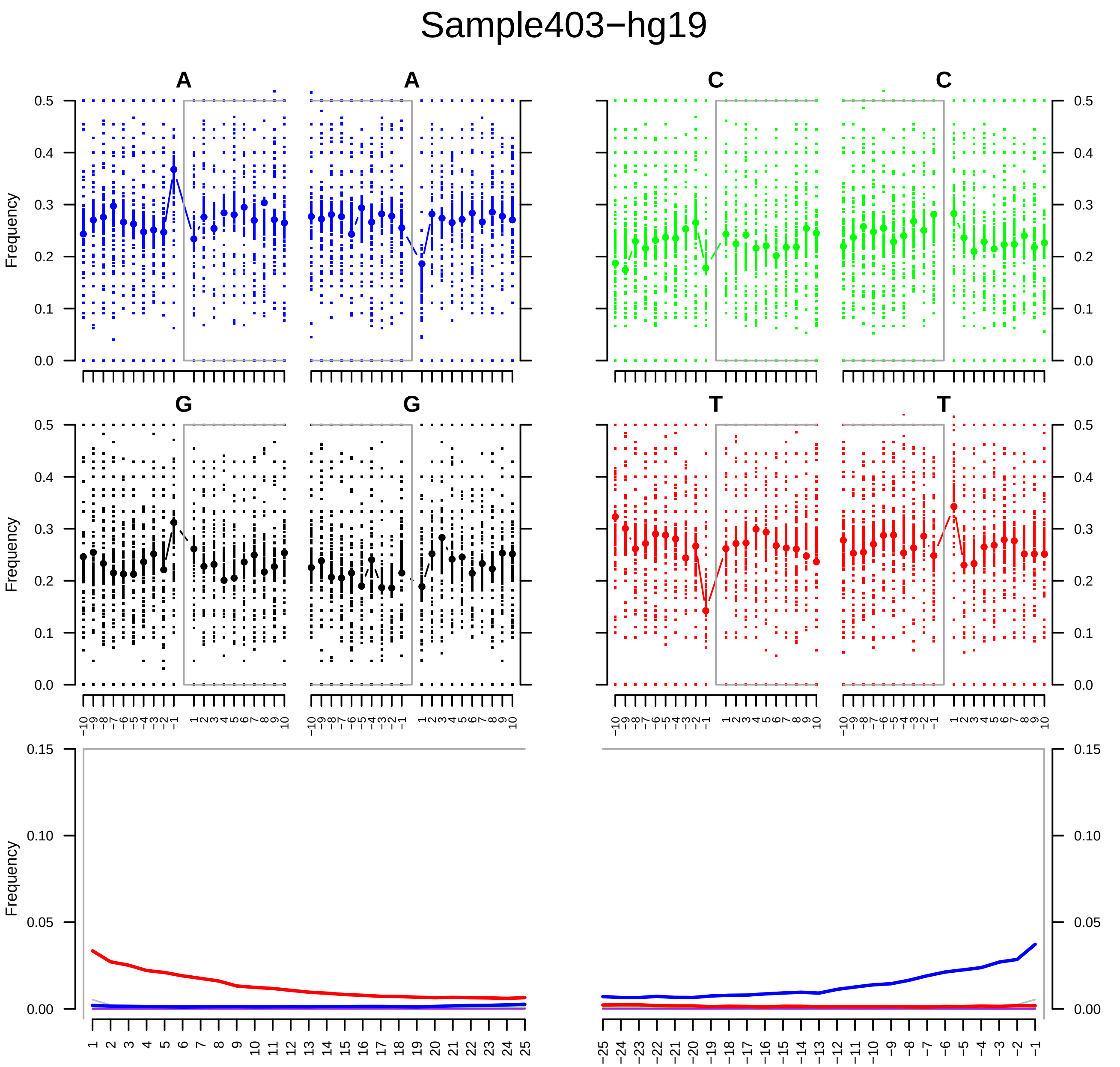

Sample403−hg19
A
A
C
C
G
G
T
T
Frequency
Frequency
Frequency

## Sample406−hg19

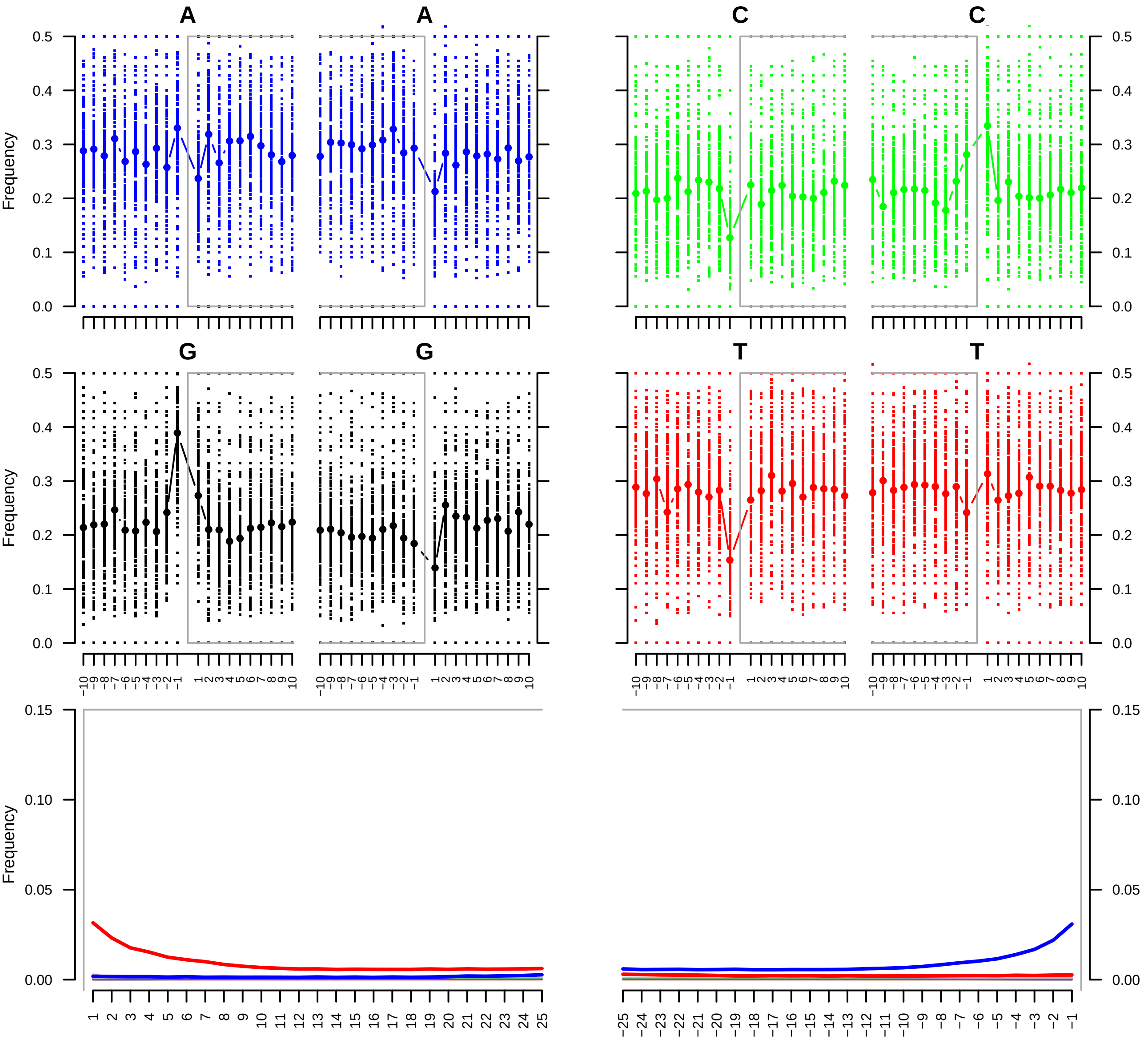

Sample702−hg19

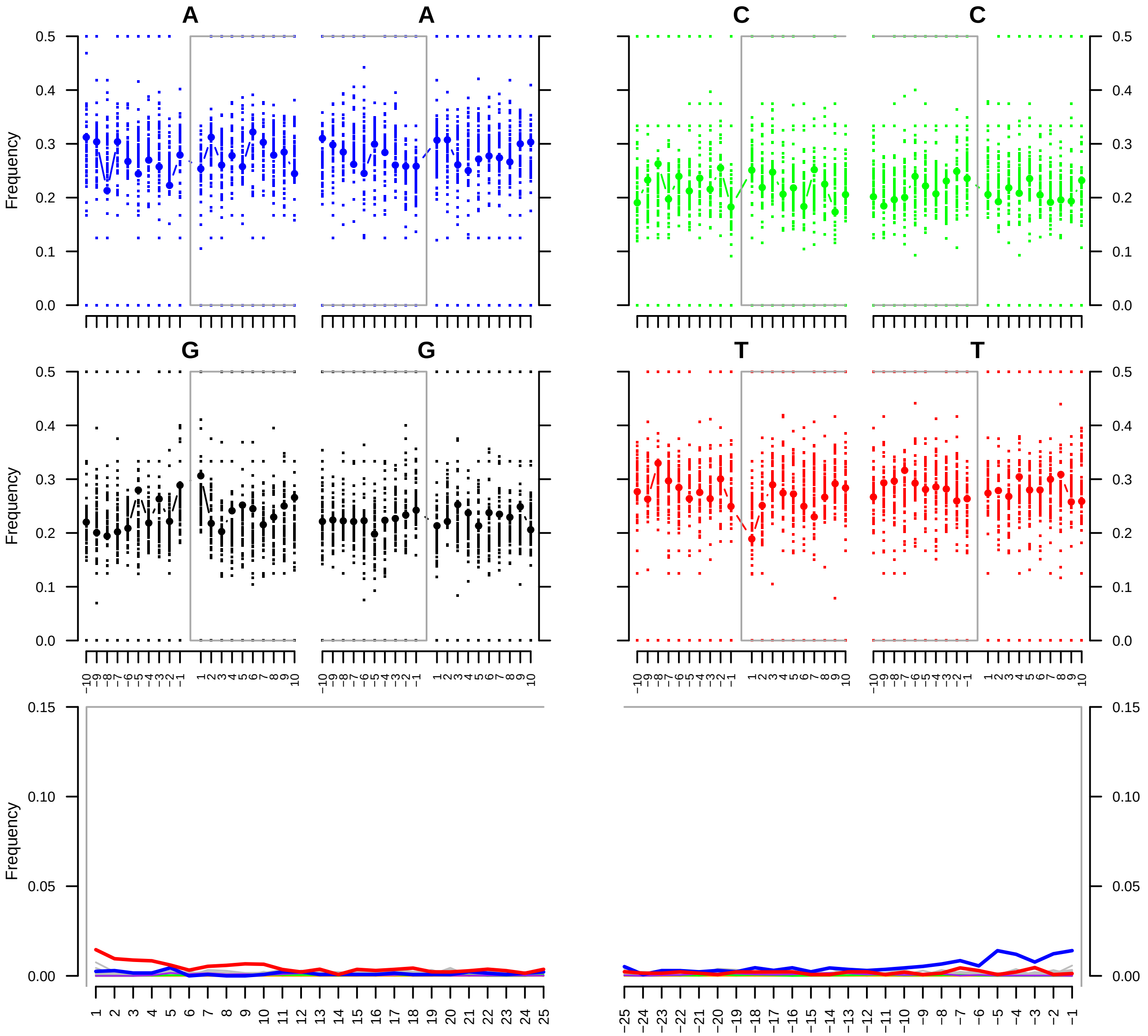

Sample306−Olsenella_uli

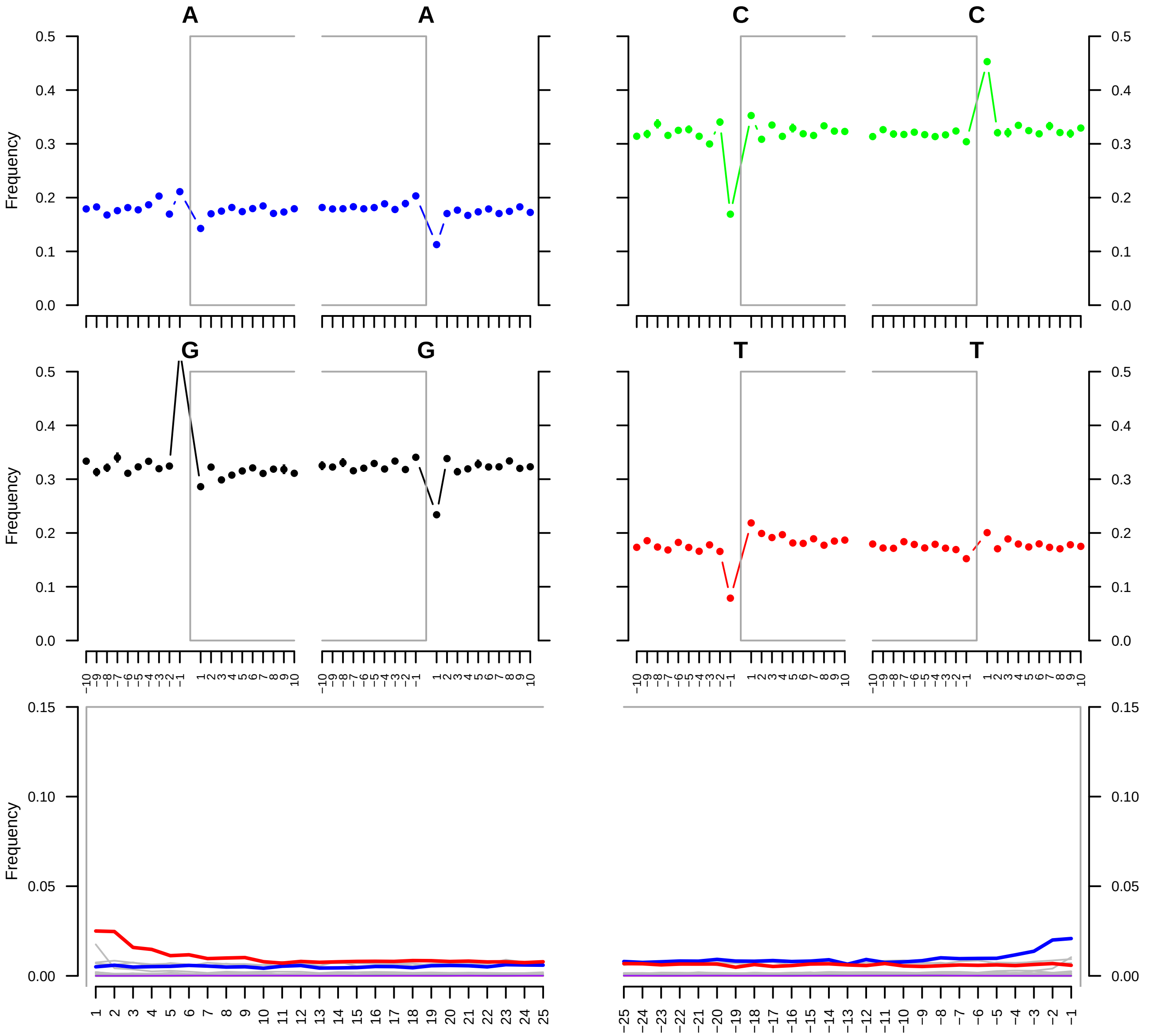

# Sample213−Pseudoramibacter_alactolyticus

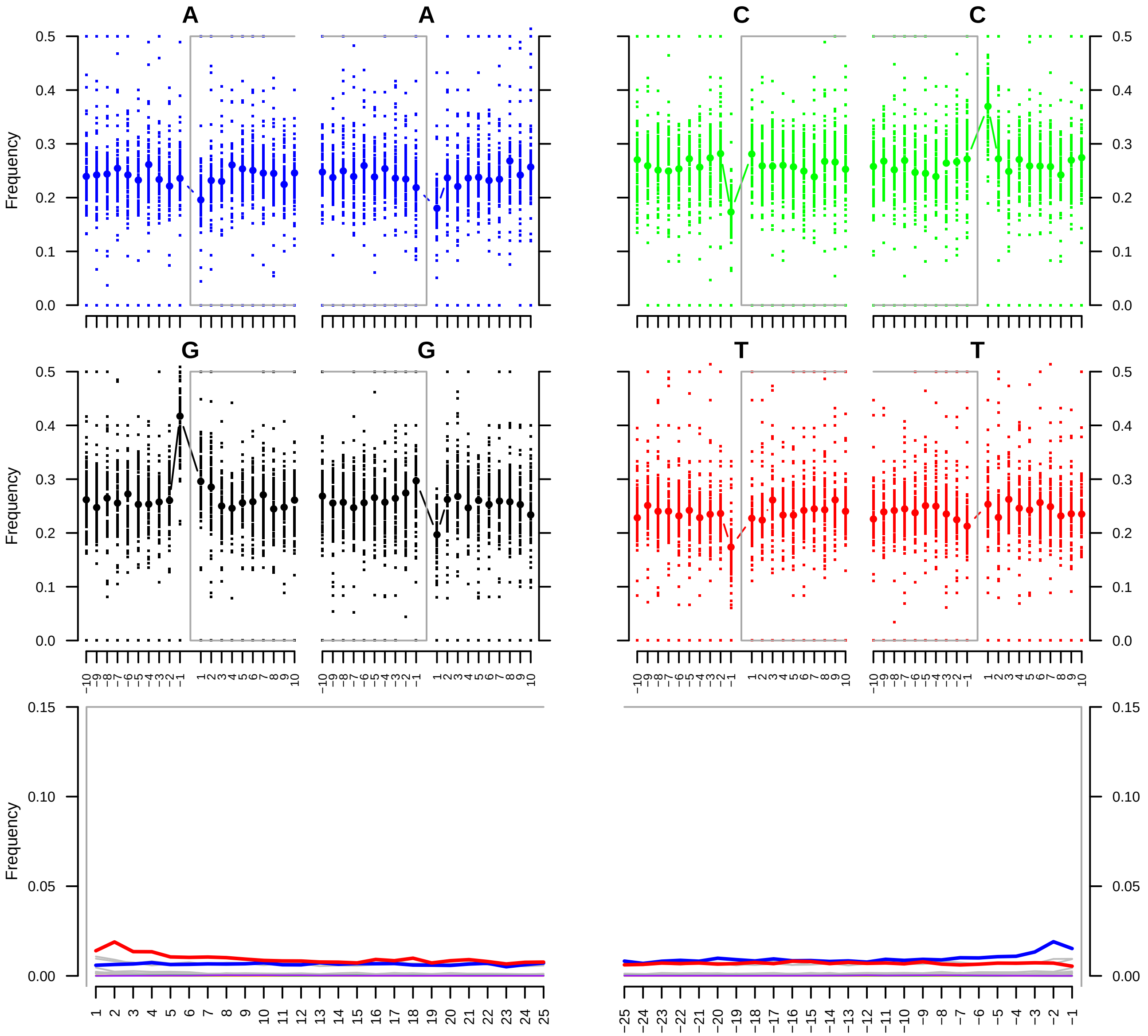

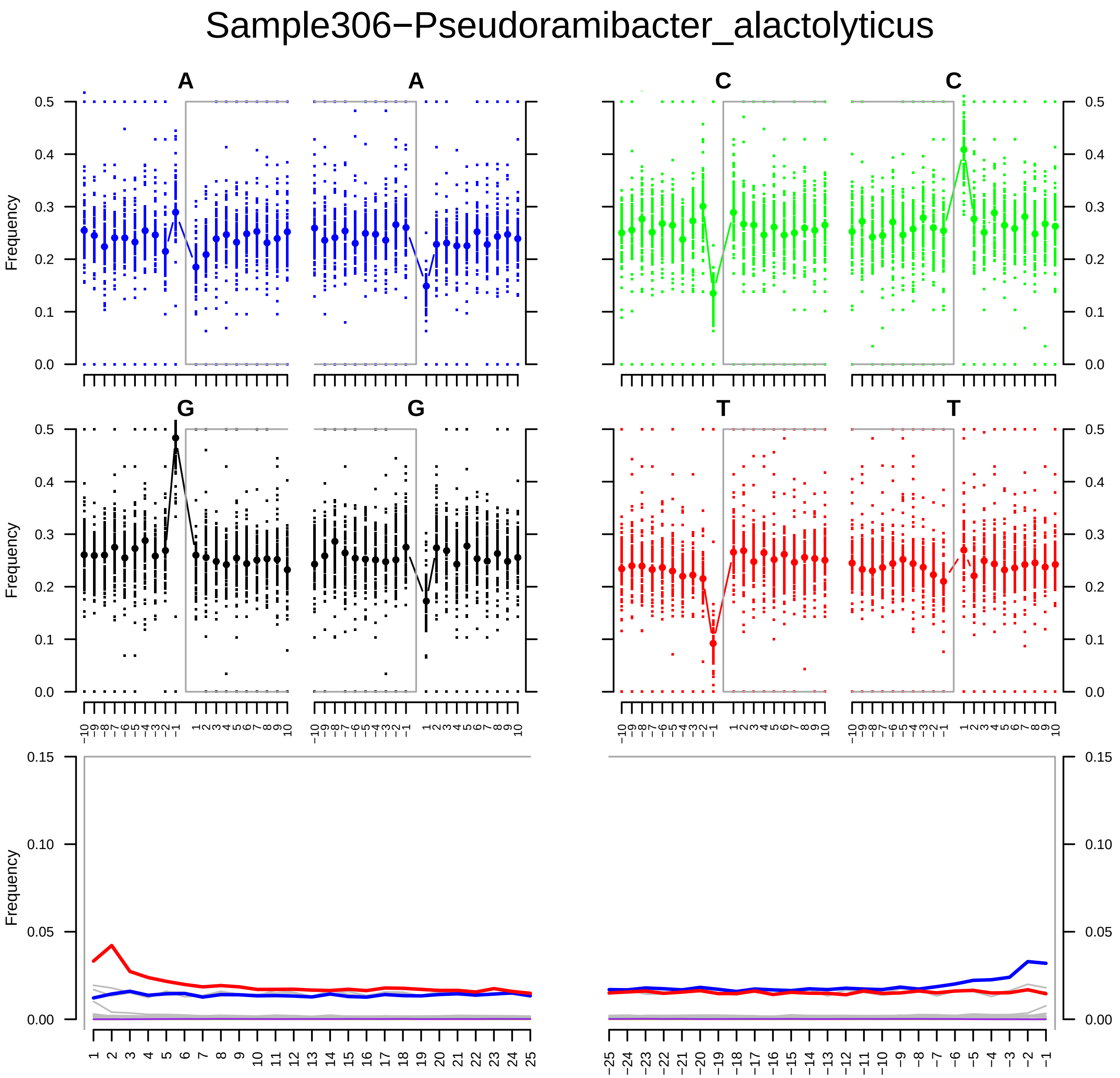
Sample306−Pseudoramibacter_alactolyticus
A
A
C
C
G
G
T
T
Frequency
Frequency
Frequency
0.0
0.1
0.2
0.3
0.4
0.5
0.00
0.05
0.10
0.15

# Sample406−Rothia_dentocariosa

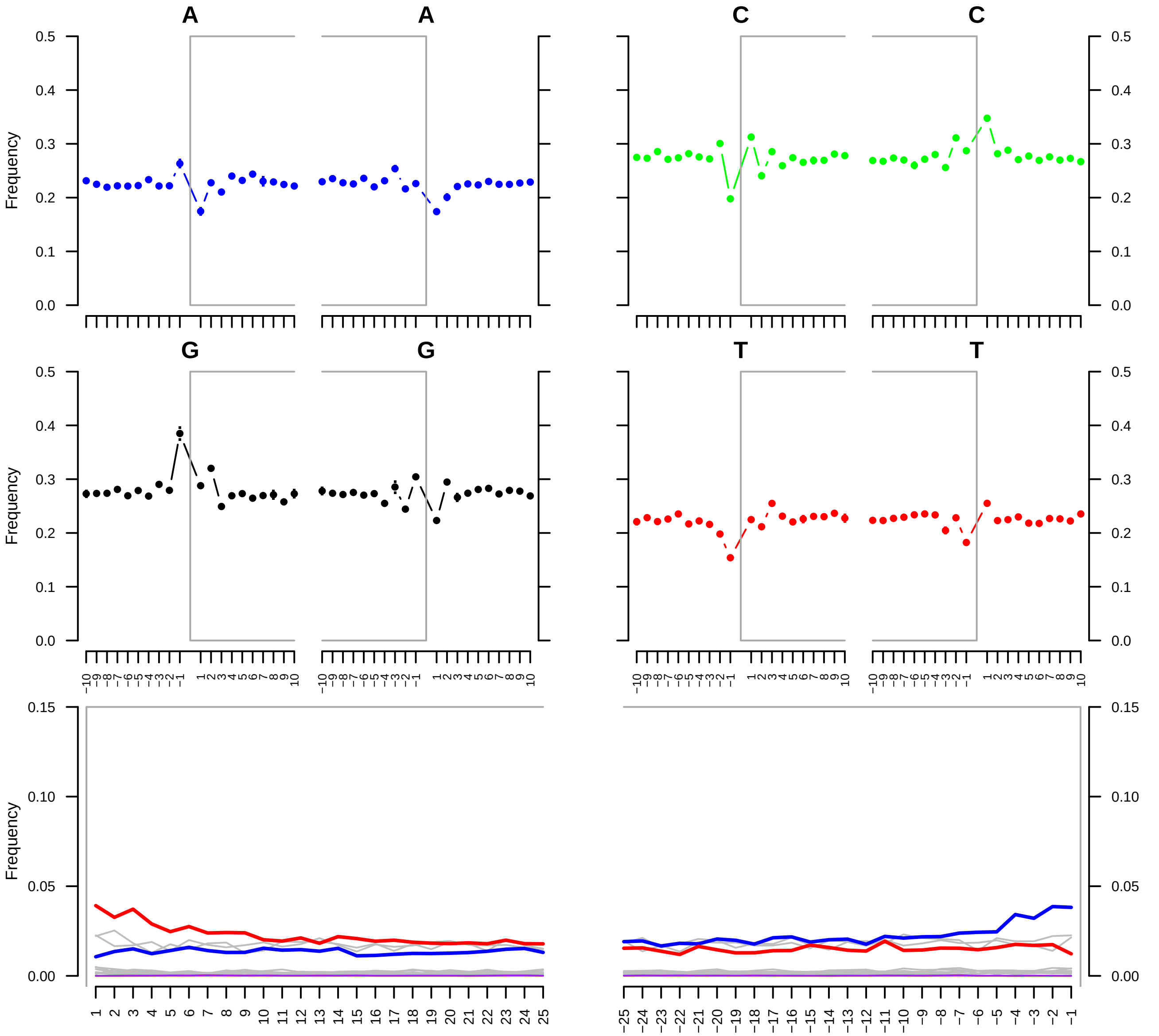

# Sample213−Tannerella_forsythia

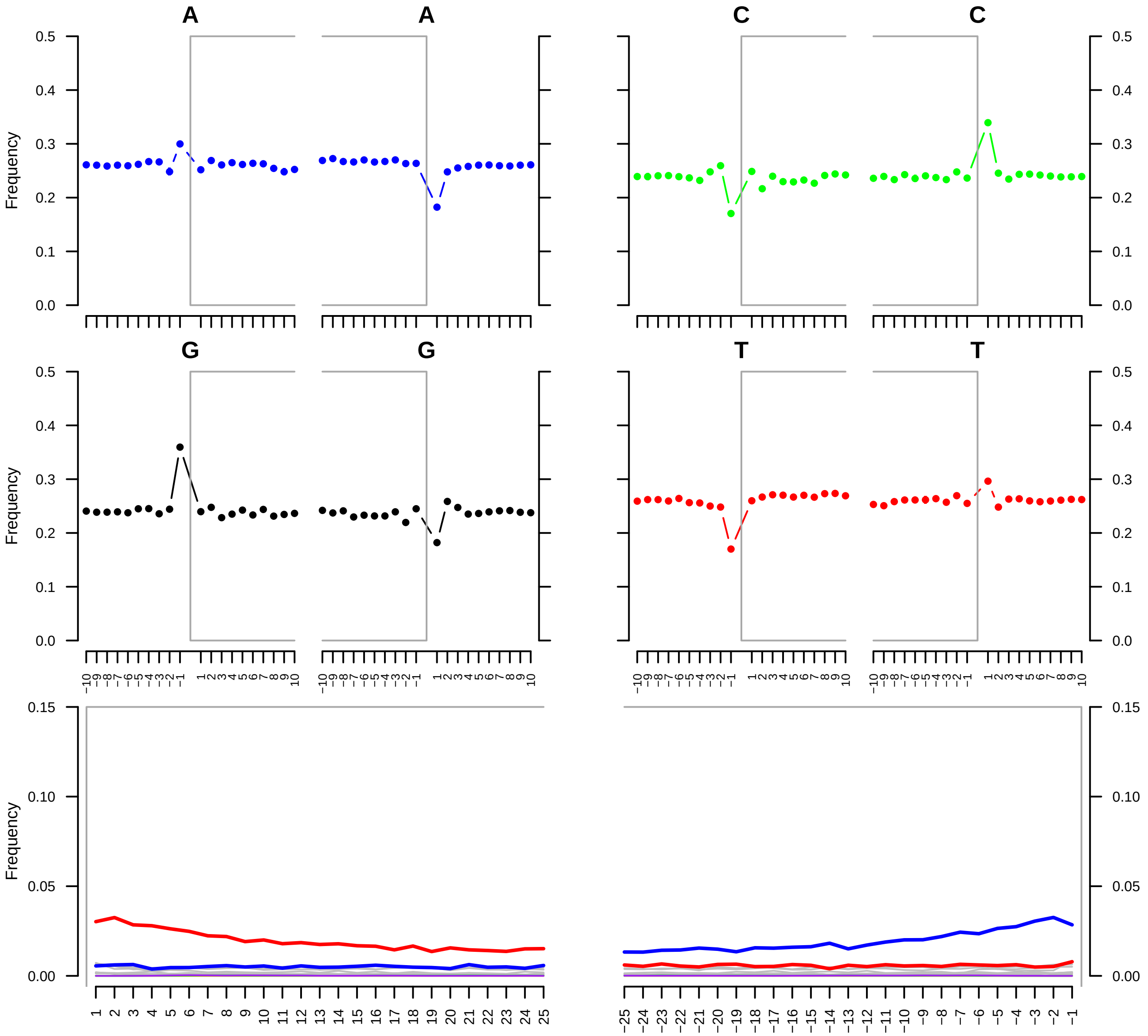

# Sample306−Tannerella_forsythia

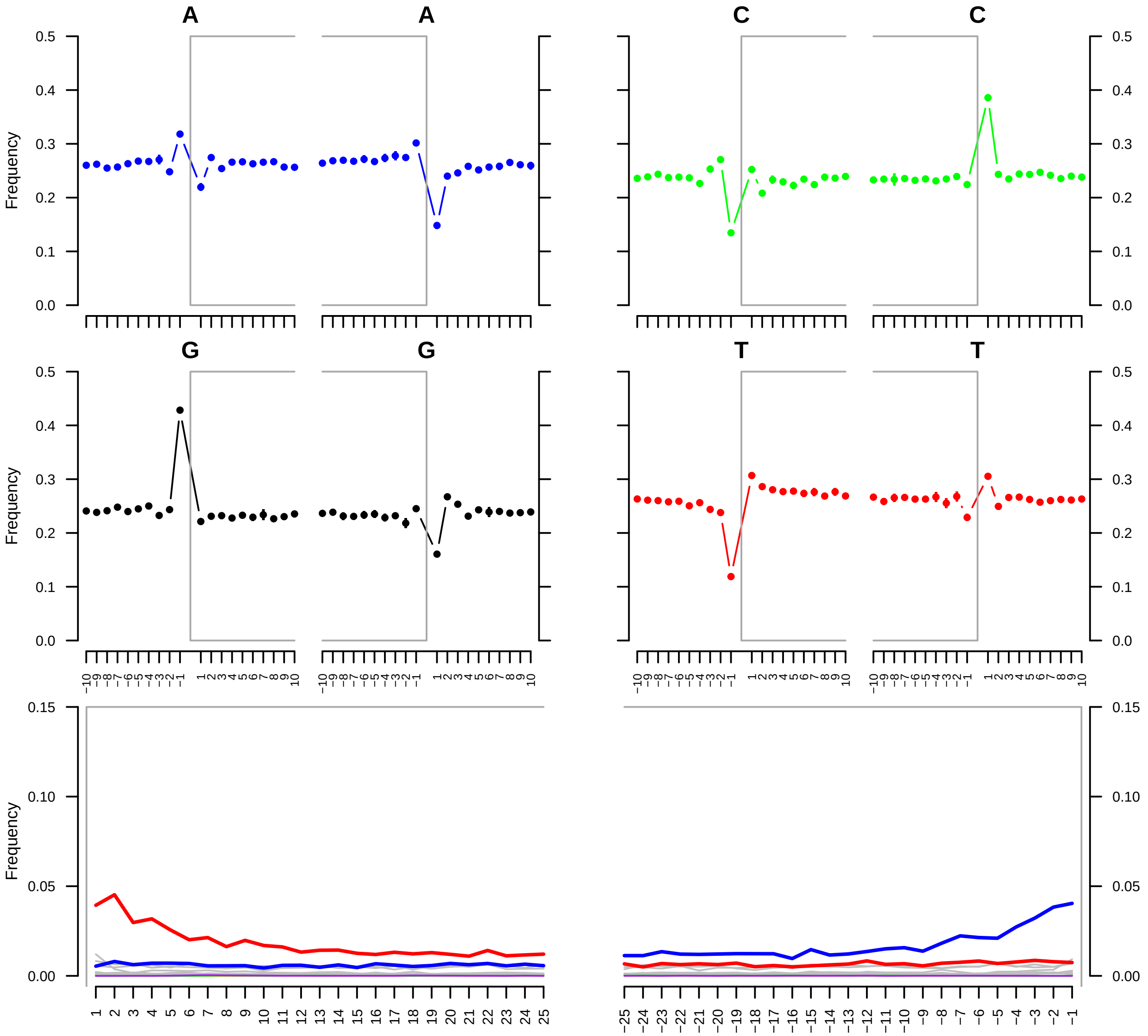

Supplement: S2 Appendix — (PDF) [file pone.0196482.s008.pdf]
